# Educational policies can strengthen climate coalitions

**DOI:** 10.1073/pnas.2533821123

**Published:** 2026-05-14

**Authors:** Max Bradley, Rens Chazottes, Susanna Garside, Nina Lopez-Uroz

**Affiliations:** ^a^ Department of Political and Social Sciences, European University Institute, Fiesole 50014, FI, Italy; ^b^ Swiss Federal Institute of Technology Lausanne, Lausanne 1015, Switzerland; ^c^ Department of Politics and International Relations, University of Oxford, Oxford OX1 3UQ, United Kingdom; ^d^ Max Planck Institute for the Study of Societies, Cologne 50676, Germany

**Keywords:** education, climate policy, political preferences, field experiment

## Abstract

Building public support for ambitious climate policies is a central challenge for governments seeking to decarbonize their economies. Many climate change mitigation policies impose visible material costs on citizens, and governments have limited fiscal capacity to deploy compensatory measures. Education is often used as a tool to build public awareness about climate change, but its effect on support for climate policies is not well understood. We evaluate the effects of a climate education policy through the study of a real-world large-scale educational intervention: a 3-h interactive workshop which has so far been implemented in over 500 French universities. We employed a randomized control trial reaching 1,845 students across 167 workshops. Students who took the workshop expressed 7 percentage points higher support for costly climate policies, including a beef tax, short-haul flight ban, and meat-free university canteen, compared with the control group. The workshop increased beliefs in the effectiveness of these policies and elicited more positive emotions about climate action. Evidence from a subsample of follow-up survey respondents suggests these effects may persist for at least 6 wk. We find no evidence that the workshop increases willingness to donate to a climate NGO. Overall, the results suggest that well-designed climate education can play a role in broadening public coalitions for ambitious climate policies by strengthening perceived policy effectiveness and support for costly policies.

## Significance

Education is widely used as a tool to build public support for climate action, yet little evidence exists about its effectiveness. We show in a large-scale field experiment that a brief, interactive climate workshop increases support for costly climate policies, such as meat taxes and flight restrictions. The effects are driven by stronger beliefs in policy effectiveness and more positive emotions toward climate action. Our findings suggest that well-designed educational programs can have a role to play in broadening coalitions for climate policies.

Governments worldwide face the challenge of building public support for climate policies that require significant personal sacrifice. These costly climate policies—such as meat taxes or flight restrictions—require large-scale behavioral change and impose visible, concentrated costs on individuals while providing diffuse social benefits (1, 2). So far, the literature has concentrated on compensation as a means to build broader coalitions of support for climate policies (2–4). However, given lackluster economic growth and worsening budgetary pressures (5), many governments are constrained in their use of fiscal incentives and thus leverage an additional coalition-building strategy: climate education.

This emphasis on climate education is reflected in Article 12 of the Paris Agreement, which calls for enhanced climate change education, training, and public awareness (6). Many governments have heeded this call and adopted climate education programs. For instance, the Italian government passed legislation in 2019 to make climate change study mandatory in schools (7), Argentina requires that all public employees receive environmental training (8), and several U.S. states have recently passed laws to incorporate climate change education into school curricula (9).

Despite growing political enthusiasm for climate education, the evidence on its effectiveness is mixed. Meta-analyses of field interventions find education to be one of the least effective strategies for promoting proenvironmental behaviors (10, 11). A global megastudy conducted in 63 countries found similarly muted effects on climate policy support across eleven different informational interventions (12). A review of school-based programs finds large gains in knowledge, but only small and inconsistent shifts in climate attitudes (13). Is climate education an effective tool for building political coalitions in favor of addressing climate change?

We suggest that the mixed findings in prior research reflect the conditions under which most climate educational interventions have been designed and tested, rather than the inherent effectiveness of education itself. Researchers have primarily studied climate education in isolated laboratory or survey experimental settings (12), in field interventions that provided abstract rather than personally relevant information (14), and that adopted a passive approach to learning. In education research, active learning, which engages students more directly in the learning process, has been shown to be an effective technique to improve student performance (15) and narrow achievement gaps for underrepresented students (16). Relatedly, several studies show that climate educational interventions are more effective when they have certain characteristics, such as taking place in real-world settings that foster peer discussion and social comparisons (17–19), combining factual and personally relevant information in an interactive environment (20–22), and reaching audiences which remain “impressionable” and thus more open to shifting attitudes (23–25).

**Fig. 1.**
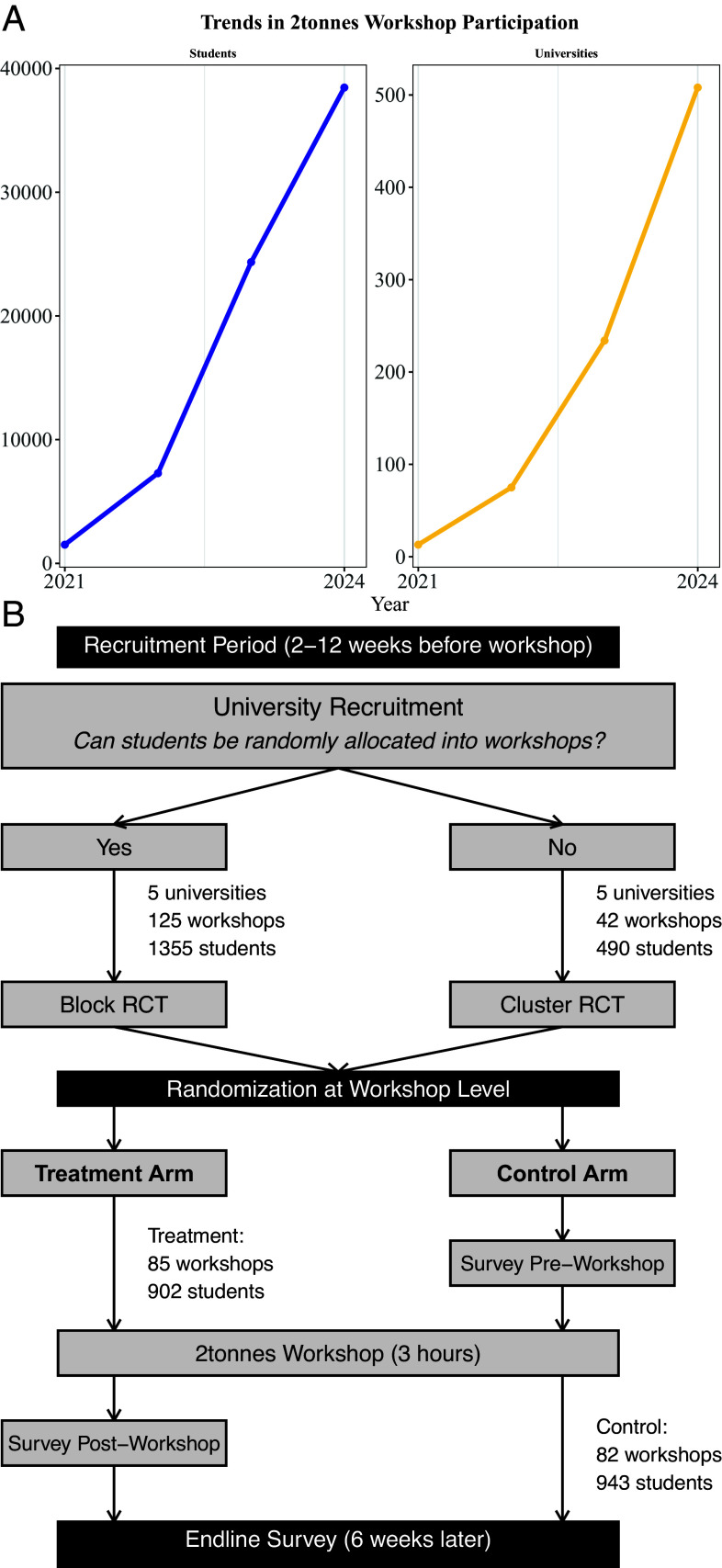
(*A*) Growth of 2tonnes participation and (*B*) research design.

Our study evaluates an educational intervention that combines such characteristics. The “2tonnes” workshop is a standardized 3-h interactive educational experience. Delivered in a real-world setting by trained facilitators, the workshop blends factual learning on effective climate change mitigation actions with a serious-game simulation in which participants use their own carbon footprint data to explore how to best reach the 2tCO2e/year per person goal by 2050. For each of the eight decision-making rounds, participants are asked to pick actions at the individual or collective level to reduce their emissions, for example, regarding transport or food. Thanks to the simulation tool, participants are told after each round how effective their choices were in terms of carbon footprint reduction and can compare their outcome with other participants. Hence, the workshop takes a science-based approach to teach about the relative effectiveness of different climate actions and blends education with social comparison aspects (see *Materials and Methods* and *SI Appendix*, section A for more information on the content of the workshop). As shown in Fig. 1*A*, there has been a rapid increase in the deployment of the 2tonnes workshop across French universities, with over 38,000 students across 500 universities participating in 2024. Universities have begun voluntarily implementing the workshop in response to policy directives from the French government recommending universities and higher education institutions to incorporate ecological and environmental education into their curricula since 2020, especially in undergraduate programs (26, 27).

This rapid expansion provides an opportunity to evaluate whether climate education can build support for costly climate policies. To do so, we partnered with 10 French universities that integrated the 2tonnes workshop into their curricula during the 2024–25 academic year. The universities span a wide range of programs and are located across France (see *SI Appendix*, section B for more information on recruitment and sample composition).

Fig. 1*B* outlines our research design. We conducted a cluster-randomized control trial where the unit of randomization was the workshop. As taking the 2tonnes workshop was mandatory and all students must participate, the only feasible source of exogenous variation is when students complete the survey. Within each university, workshops were randomly assigned to one of two conditions. Students in control workshops completed the survey immediately before the workshop began, while students in the treatment workshops completed the survey immediately after the workshop concluded. Our final sample includes 167 workshops (1,845 students) across 10 universities, with 82 workshops (943 students) randomly assigned to control and 85 workshops (902 students) randomly assigned to treatment. In five universities, students could be randomly allocated into workshop groups; in the remaining five, workshop groups were fixed by the university. In all cases, however, the workshop-level timing randomization is the source of causal identification.

Because the workshop was mandatory, participation in our survey was high, with an overall attrition rate of 19% (see *SI Appendix*, section B for more). We also observe a high compliance rate at 93%. This means that concerns about self-selection into the workshop are minimal. Randomization achieved balance across pretreatment covariates (see *SI Appendix*, section C for more). This design preserves the collective nature of the intervention while ensuring that treated and control students differ only in whether they have just completed the workshop, allowing us to identify the causal effect of participation.

Our primary outcome measures are support for costly climate policies. They are i) a tax doubling the price of beef, ii) a ban on flights for destinations accessible by train or bus within 6 h, and iii) a university-level ban on serving meat in the canteen. All outcomes are informed by previous academic studies (28) and contemporary policy debates in France (29–31). Participants rated each policy on a five-point Likert scale, which we dichotomize as 1 for those who “support” or “strongly support” the policy, and 0 otherwise (see *SI Appendix*, section D for more). As a secondary outcome, we measure willingness to donate to a climate NGO. Participants who opted in to a €100 lottery could donate a share of their potential winnings to *Réseau Action Climat*, a well-known French climate NGO.

To assess longer-term effects, we recontacted participants 6 wk after the workshop with an invitation to complete an endline survey. Participation was voluntary, yielding a final endline sample of 330 respondents (18%). Endline respondents differed somewhat in attitudes and behavior from nonrespondents, but importantly not by treatment status (see *SI Appendix*, section C for more).

Our preregistered hypotheses are shown in Table 1. In H1, we examine whether participation in the 2tonnes workshop increases support for costly climate policies. We also test a preregistered set of mediating, moderating, and exploratory hypotheses (see *SI Appendix*, section D for more). We conceptualize H2, which concerns willingness to donate to a climate NGO, as a behavioral extension of H1.

**Table 1.** Preregistered hypotheses

	Statement
**H1**	The workshop increases participants’ support for costly climate policies.
H1a	Driven by a decrease in psychological distance to the climate crisis.
H1b	Driven by an increase in belief in policy effectiveness.
H1c	Stronger for participants with lower personal costs.
H1d	Weaker for participants with low trust in government.
**H2**	The workshop increases willingness to donate to a climate NGO that supports the proposed policy goals.
H2a	Stronger when the NGO is framed as advocating on the supported issue.

## Results

We test our primary, secondary, and exploratory hypotheses using ordinary least squares regression with SE clustered at the workshop level to estimate the intent-to-treat (ITT) effect of participating in the workshop, in line with our preanalysis plan. All regressions include university and experimental block fixed effects. For robustness, we also estimate the local average treatment effect (LATE) and find results consistent with the main analysis (*SI Appendix*, section F.3). All further results not reported below can be found in *SI Appendix*, sections E and H.

### Main Effect: Support for Costly Climate Policies.

**Fig. 2.**
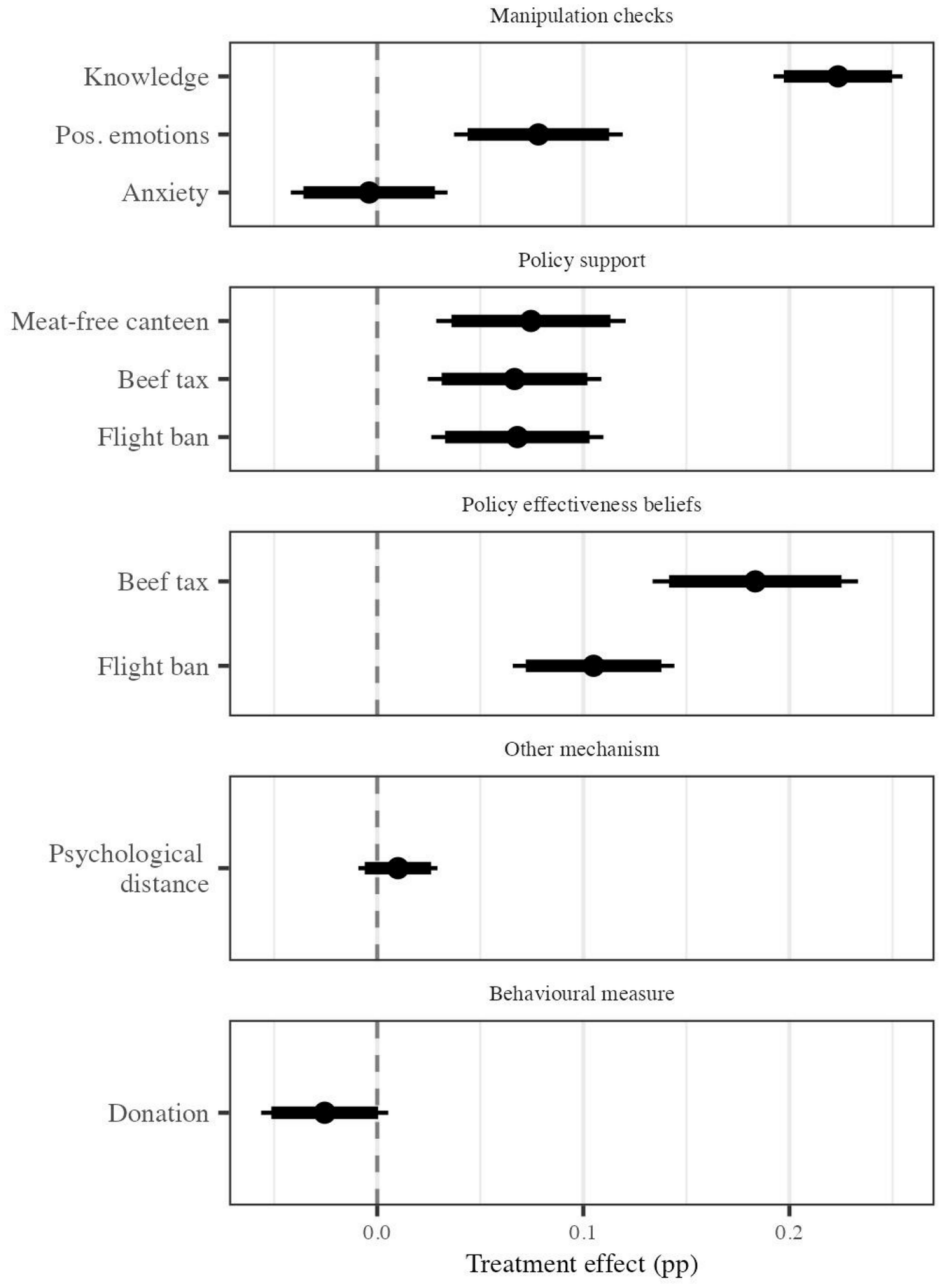
Results for models testing H1, H1a, H1b, and H2. All models use an ITT estimator with experimental block fixed effects and cluster robust SE at the workshop level. Thick (thin) bars represent 95% (90%) CI.

Our results show that participating in the 2tonnes workshop increases support for costly climate policies (H1). The results, presented in Fig. 2, show that across each of the three policy outcomes, support was 7 percentage points higher among students surveyed after the workshop compared to those surveyed before.

Breaking the results down by policy, we observe that support for a tax doubling the price of beef increases by 6.7 percentage points from a baseline of 34% (*β* = 0.07; *P* = 0.004). Support for a ban on short-haul flights rose by 6.7 percentage points from a baseline of 55% (*β* = 0.07; *P* = 0.003). Likewise, support for a university-level ban on serving meat in canteens increases by 6.8 percentage points from a baseline of 36% (*β* = 0.07; *P* = 0.003). These results provide robust evidence that participating in the 2tonnes workshop positively shifts attitudes toward costly climate policies.

### Mechanisms.

We preregistered two main mediators—that increased policy support is driven by i) a decrease in the psychological distance between a participant and the climate (H1a) and ii) an increase in participants’ belief in the effectiveness of the policy (H1b).

We report the results of a causal mediation analysis (32). As detailed in *SI Appendix*, section E.3, we compute the average causal mediation effect (ACME), the average direct effect (ADE), the total effect, and the proportion mediated. Table 2 reports the results. For both beef tax support and flight ban support, the indirect effect through psychological distance was small and not statistically different from zero (ACMEbeef=0.002, 95% CI [−0.005, 0.009]; ACMEflight=0.001, 95% CI [−0.004, 0.007]), whereas the indirect effect through perceived effectiveness was positive and statistically significant (ACMEbeef=0.067, 95% CI [0.051, 0.084]; ACMEflight=0.052, 95% CI [0.033, 0.072]). The direct effect remained significant for psychological distance but not for effectiveness, indicating that perceived effectiveness accounted for most of the total effect, whereas psychological distance explained little of the treatment impact. Sensitivity analyses are reported in *SI Appendix*, Table S14. Overall, these findings suggest that changes in perceived policy effectiveness, rather than shifts in psychological distance, primarily drove increases in policy support.

**Table 2.** Causal mediation analysis of workshop effects

	Beef Tax Support		Flight Ban Support	
	Psych. Distance	Effectiveness	Psych. Distance	Effectiveness
ACME	0.002 [−0.005, 0.009]	0.067*** [0.051, 0.084]	0.001 [−0.004, 0.007]	0.052*** [0.033, 0.072]
ADE	0.051*** [0.022, 0.081]	−0.015 [−0.040, 0.012]	0.053*** [0.022, 0.084]	0.002 [−0.023, 0.028]
Total effect	0.053*** [0.025, 0.084]	0.052*** [0.025, 0.084]	0.054*** [0.024, 0.086]	0.054*** [0.025, 0.086]

This analysis is supported by additional analyses in Fig. 2 and *SI Appendix*, section E.3, which reports each mediation pathway as an intermediate outcome using the same regression framework as we do for our main outcomes.

We also preregistered two manipulation checks that can be understood as mechanisms (Fig. 2 and *SI Appendix*, section E.3). The first dimension is information uptake, as measured by two multiple-choice knowledge questions about the components of the average French carbon footprint. Taking the average of these two variables as our outcome variable, we find that the workshop significantly increases respondents’ knowledge about the sources of greenhouse gas emissions (β=0.22; *P* < 0.001).

The second dimension is participants’ emotions when thinking about climate change. We presented respondents with a choice list of different emotions they could feel in reaction to climate change (in which they could choose up to two emotions), from which we created a binary variable indicating whether they picked at least one positive emotion from the list. The results indicate that taking the 2tonnes workshop increases positive emotions among participants, i.e., hope, calm, optimism, and motivation (β=0.08; *P* < 0.001).

The results for these two dimensions are clear—taking the 2tonnes workshop increases participants’ knowledge about what causes climate change as well as their positivity toward the issue.

### Heterogeneous Treatment Effects.

**Fig. 3.**
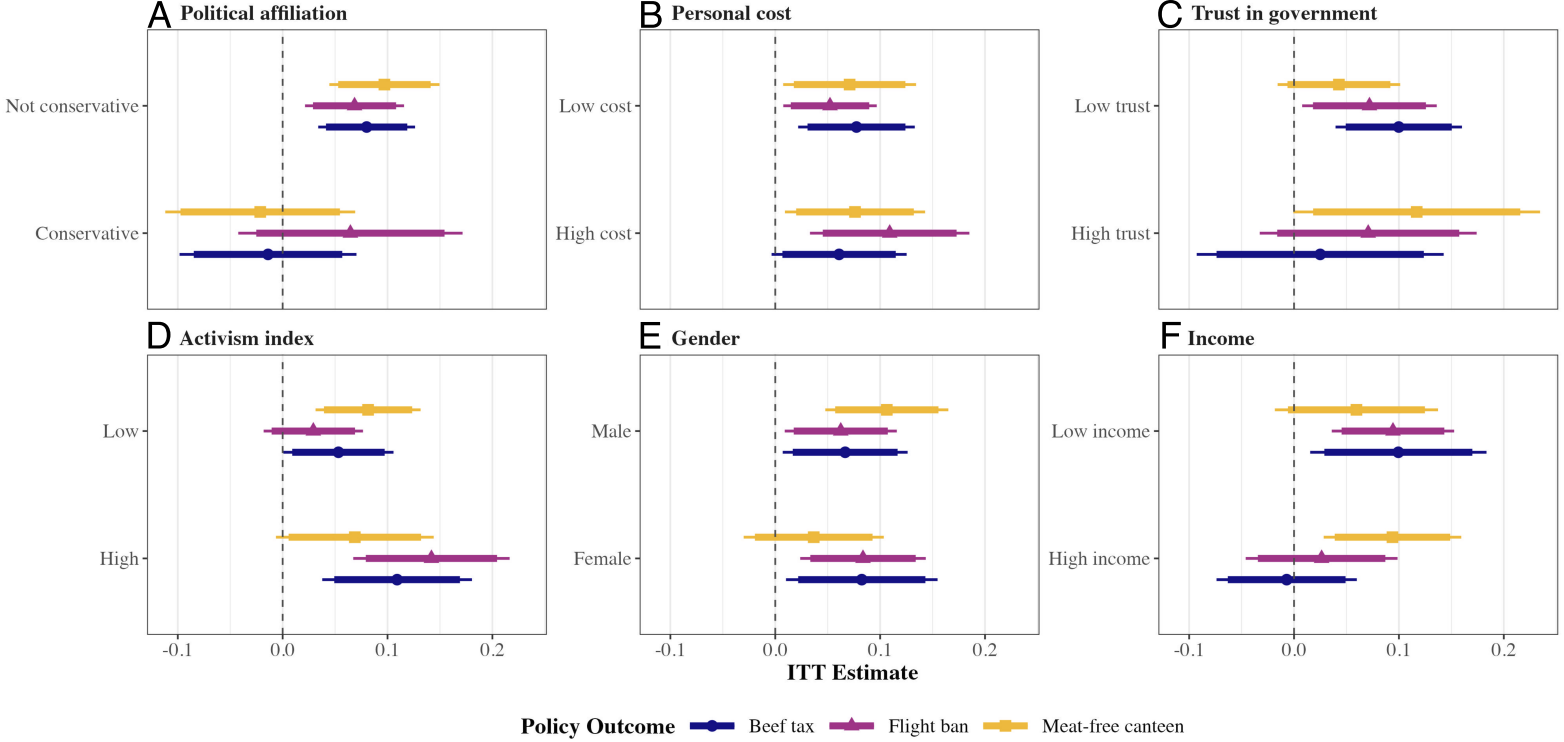
Results for models testing heterogeneous treatment effects with political affiliation, personal cost, trust in government, activism, gender, and income as moderators. See *SI Appendix*, section D.3 for more information on how we measured these variables. All models use an ITT estimator with experimental block fixed effects and cluster robust SE at the workshop level. Thick (thin) bars represent 95% (90%) CI. These results only include data on the workshop day, not the 6-wk follow-up survey.

We preregistered several exploratory moderators (*SI Appendix*, section D.3). All results are presented in Fig. 3. Each moderator is tested using the same regression framework as the main effects, but including an interaction term for the relevant variable. We report the *β* from the interaction terms here, with the full tables provided in *SI Appendix*, section E.4.

These results should be interpreted as exploratory and descriptive for two reasons. First, because these moderators are not randomly assigned, the interaction estimates may reflect spurious relationships. They should therefore not be interpreted as causal heterogeneous treatment effects, but rather as describing how experimentally identified average treatment effects vary across pretreatment characteristics. Second, statistical power to detect heterogeneous effects is limited. As discussed in *SI Appendix*, Table S27, under conservative assumptions, the minimum detectable subgroup differences are approximately 9 to 10 percentage points. Smaller interaction effects may therefore go undetected.

First, we expected weaker effects among participants who face a higher level of personal cost from the proposed policies (H1c). Personal cost is measured in terms of both meat and flight consumption, depending on the outcome (*SI Appendix*, section D.3). Contrary to our expectations, we observe no difference in treatment effects between participants facing higher and lower personal costs. All treatment effects are positive and significant across all policy outcomes regardless of the cost faced (Fig. 3*B*).

Second, we expected effects to be weaker among participants with low trust in government (H1d), measured by the degree to which respondents perceived French institutions as corrupt. Again, our expectations were not borne out (Fig. 3*C*). While treatment effects were larger among low trust respondents for both the beef tax and the flight ban, the interaction terms were not significant (*β* = 0.07; *P* = 0.07 and β=−0.01; *P* = 0.89). These results indicate that the effectiveness of the workshop is not undermined by low levels of institutional trust.

The lack of heterogeneity across genders is noteworthy (Fig. 3*E*), especially given recent research emphasizing a gender gap in climate attitudes (33, 34). If anything, the workshop seems to have a stronger effect on men compared to women on support for a meat-free canteen, although the interaction is not significant at conventional levels (β=−0.07; *P* = 0.12). Additionally, we observe that the workshop was somewhat more effective for respondents from low income families (Fig. 3*F*) who are more supportive of both a ban on beef and flight restrictions, although again with muted effects for the interaction terms (β=−0.09; *P* = 0.10 and β=−0.05; *P* = 0.28).

Finally, participants who reported feeling closest to a center-right or right-wing party appeared less responsive to the workshop compared to more moderate or left-wing participants (Fig. 3*A*), although only the interaction term for the meat-free canteen is significant (β=−0.11; *P* = 0.04). Additionally, participants with previous climate activism experience exhibit stronger responses to the workshop (Fig. 3*D*), especially in their support for a short-haul flight ban, relative to participants without such experience (*β* = 0.09; *P* = 0.03).

### Persistence of Effects.

For the subsample of respondents who answered our endline survey, we test whether the observed effects persist 6 wk after the 2tonnes workshop date. Respondents in this subsample are on average, slightly older and come from less wealthy families. They are more left-wing, fly less often, and eat less red meat. In line with this demographic profile, these respondents are also more likely to show greater support for our three main climate policy outcome variables (*SI Appendix*, Table S7). We therefore interpret these results as suggestive evidence of the persistence of workshop effects.

**Fig. 4.**
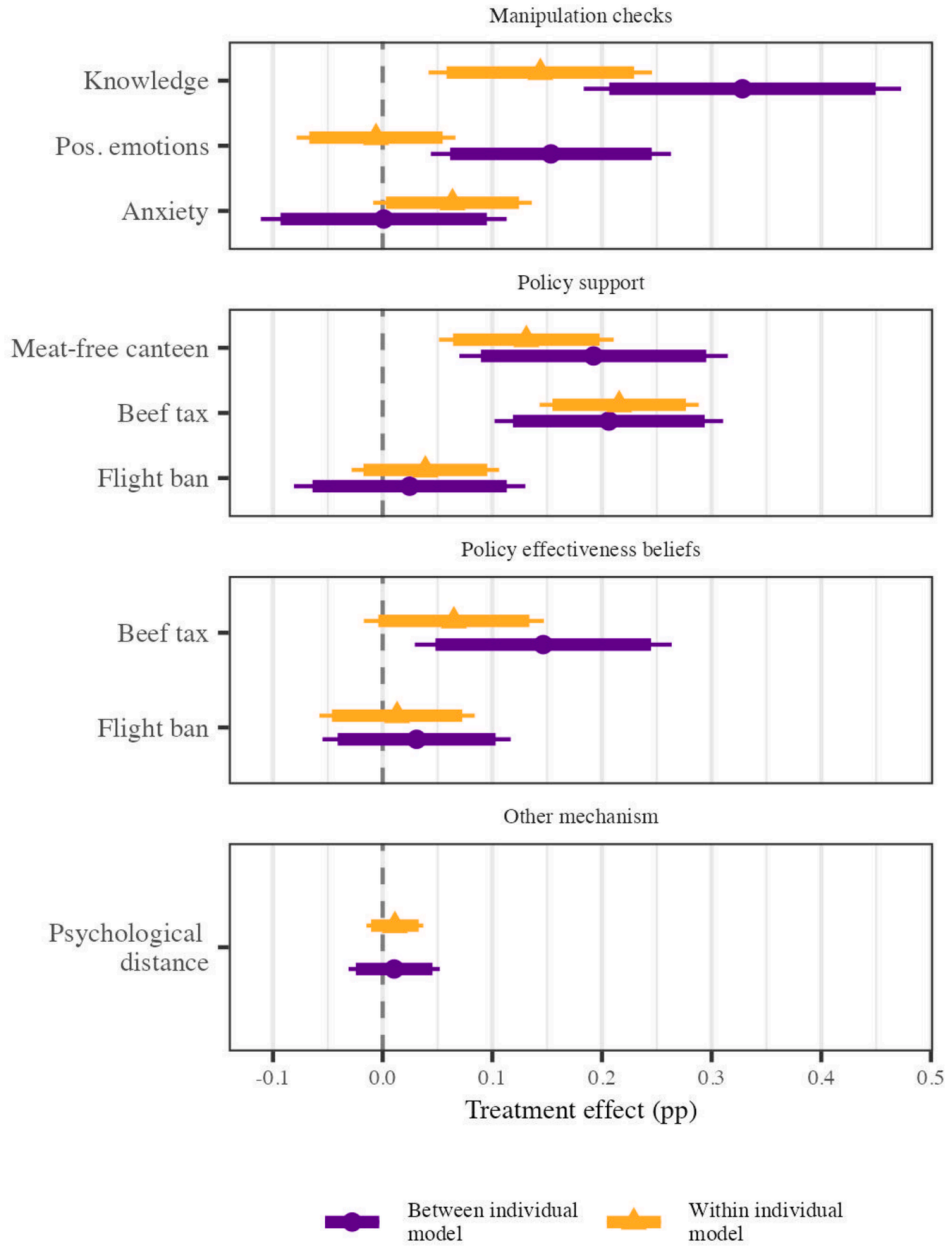
Estimated treatment effects 6 wk after the workshop using within-group (orange triangle) and between-group (purple dot) designs. All models use an ITT estimator with experimental block fixed effects and cluster robust SE at the workshop level. Thick (thin) bars represent 95% (90%) CI. All variables are measured in the same way across both surveys, with the exception of knowledge, which is based on two questions in the initial survey but only one of these two questions at the endline (*SI Appendix*, section D.2).

We employ two complementary estimation strategies. First, a within-individual estimator compares students who were initially in the control group at baselines but later completed the endline survey after completing the workshop (see *SI Appendix*, section E.1 for more). These results, again presented in Fig. 4, show a sustained 22 percentage point increase in support for a beef tax (*β* = 0.22; *P* < 0.001) and a 13 percentage point increase in support for a meat-free university canteen policy (*β* = 0.13; *P* = 0.002). The effect on support for a flight ban was smaller and not statistically significant (*β* = 0.04; *P* = 0.50). This likely reflects ceiling effects given this outcome had the highest baseline level of support among the control group.

Second, a between-individual estimator—a difference-in-differences specification which interacts treatment status and survey wave—yields a similar pattern. Support for a beef tax increases by 20 percentage points (*β* = 0.20; *P* < 0.001), and support for a meat-free canteen policy increased by 19 percentage points (*β* = 0.19; *P* = 0.003), while the flight ban again showed no detectable effect (*β* = 0.03; *P* = 0.57). The treatment effects identified in these analyses are large, but with relatively large CI due to our small sample. However, taken together, these results provide suggestive evidence that the workshop’s effect persists over time (6-wk posttreatment) for two of the three costly climate policies, buttressing further support for **H1**.

In line with these results, we also find that several attitudinal mechanisms show persistence 6 wk after treatment. Specifically, beliefs in the effectiveness of the beef tax remain significantly higher (within *β* = 0.07, *P* = 0.05; between *β* = 0.15, *P* = 0.02), whereas beliefs in the effectiveness of the flight ban do not (within *β* = 0.01, *P* = 0.73; between *β* = 0.03, *P* = 0.45). Likewise, the workshop’s positive effects on climate knowledge (within *β* = 0.14, *P* = 0.01; between *β* = 0.33; *P* < 0.001) and on positive emotions toward climate change (within β=−0.01, *P* = 0.86; between *β* = 0.15; *P* = 0.01) both persist over the 6-wk period.

### Other Results and Robustness.

We preregistered two additional exploratory outcomes. We expected that taking the workshop would increase the salience of the climate crisis vis-Ã -vis other political issues. However, we find no meaningful effect (*β* = 0.02; *P* = 0.35; see *SI Appendix*, Table S29).

We also investigated whether participation in the workshop altered respondents’ perceptions of which actors—individuals, firms, or government—are currently doing enough to address climate change. The results, presented in *SI Appendix*, Table S30, suggest that the workshop made participants less likely to believe that individuals are doing enough (β=−0.35; *P* < 0.001) and more likely to believe that the government is doing enough (*β* = 0.22; *P* = 0.04). We find null effects for firms. That said, the control means were 4.82 for citizens (on an 11-point scale) and 3.64 for the government. This implies that, even after taking the workshop, respondents still felt the government should be doing more compared to individuals. In *SI Appendix*, we also present results for heterogeneity by university (*SI Appendix*, section E.6 and Fig. S3) and by academic discipline (*SI Appendix*, section E.6 and Fig. S4).

We conduct robustness checks re-estimating the main effects. Results using the full ordinal scale (*SI Appendix*, section F.1), a ordered logit estimator (*SI Appendix*, section F.2) and a LATE estimator confirm our findings (*SI Appendix*, section F.3). We find no evidence to suggest a confounding influence of time of day the workshop was taken (*SI Appendix*, section F.4). We also find no indication that our results are driven by any single university (*SI Appendix*, section F.5).

### Behavioral Effect.

Finally, we turn to our behavioral outcome—willingness to donate to the climate NGO, Réseau Action Climat (**H2)**. First, participants were given the opportunity to opt into a lottery to win €100, and 83% did so. They were then offered a chance to donate some or all of the potential winnings to the NGO. Fig. 2*C* shows that students in the treatment group donated, on average, €2.55 less than those in the control group, a difference that was not statistically significant (β=−2.55; *P* = 0.10). The average donation in the control group was €32. This evidence indicates that, while the workshop did increase support for costly climate policies, it did not translate into greater willingness to donate to climate advocacy groups.

Additionally, we embedded a vignette experiment in the donation task (see *SI Appendix*, section H for description and tables). Respondents were randomly assigned one of three descriptions of the NGO, i) a general description, ii) a description emphasizing the NGO’s focus on reducing beef consumption, and iii) a description emphasizing the NGO’s focus on reducing air travel. Compared to the general description, donations were €3.20 lower when the NGO was framed around beef consumption (β=−3.20; *P* = 0.08) and €1.37 lower when framed around air travel (β=−1.37; *P* = 0.50). In sum, these results do not offer support for **H2**.

## Discussion

Governments around the world are turning to climate education as a low-cost tool to build political support in favor of climate action. However, evidence of its effectiveness remains rather weak. We suggest that such weakness reflects the limitations of previous research designs, rather than the actual efficacy of climate education. By conducting a randomized control trial of a climate education program that takes place in a real-world classroom setting, blends factual with personally relevant information, fosters collaboration between participants, and targets an impressionable student audience, we provide causal evidence that climate education can increase support for costly climate policies.

Our results show that participation in the 2tonnes workshop increases support for each of the three costly climate policies we examined, with an average increase of 7 percentage points. In the endline survey completed 6 wk later by a smaller subsample, these effects persist for both a beef tax and a meat-free canteen, though not for a short-haul flight ban, where baseline support was already high. By contrast, we find no evidence that the workshop increases participants’ willingness to donate to a climate NGO, suggesting that its impact is stronger on policy attitudes than on private behavior. However, this lack of effect might be related to participants estimating their chances of winning to be low in our lottery-based design.

Analyses of mechanisms indicate that the workshop strengthens beliefs in the effectiveness of climate policies. This echoes findings from previous studies over the role of effectiveness beliefs in building up support for policies (35–37). The workshop also increases knowledge about the sources of greenhouse gas emissions and elicited positive emotions toward climate action among participants.

Subgroup analyses show little systematic variation in effects, including for participants who would bear higher or lower personal costs. This finding challenges previous work suggesting that support for climate policies can be conditional on bearing low personal costs (38, 39). Indeed, by increasing support even among participants who personally face higher costs or express low trust in institutions, education can help expand coalitions that might otherwise resist climate action.

Our findings have direct implications for policymakers. They show that educational interventions relying on active learning can complement fiscal and regulatory instruments by creating political space for ambitious policy action. According to our estimates, the workshop has a higher cost-effectiveness ratio (cost per participant divided by its effect on policy support) than other stand-alone climate workshops and online simulations (*SI Appendix*, section A.3). Its effect is also comparatively large relative to studies with similar designs and outcomes: the benchmark effect size in our study is Hedges’ g = 0.16 (95% CI [0.09, 0.23]), whereas most informational or normative treatments typically produce effect sizes in the g = 0.02 to 0.10 range (*SI Appendix*, section G). Our findings are consistent with meta-analytic evidence from a range of policy domains showing that communicating policy effectiveness increases public support by an average of four percentage points (40).

Our study has limitations. First, while the university context accurately captures the group targeted by this third-level education policy in the French context, we take caution in assuming generalizability to broader populations. Our study population is more supportive of climate policies than the general French population: baseline support for a beef tax is 34% in our control group compared with a national average of 29% (28). Our estimates may thus represent an upper bound for what might be observed in other contexts. Second, we acknowledge that although participants were aware of the confidentiality of their responses, greater social desirability bias in survey responses among the treatment group could potentially inflate the observed treatment effects. Third, attrition at the endline reduces our ability to draw strong conclusions about the persistence of the observed effects (*SI Appendix*, section B). Future work should test similar interventions over longer periods in different institutional contexts (e.g., workplaces) where participants are older or more politically diverse. Finally, our design did not allow us to track participants’ decisions during the workshop; we were therefore only able to estimate the aggregate effect of the overall workshop experience. Future work could test which components of this bundled treatment drive changes in participants’ policy preferences and effectiveness beliefs.

Overall, our findings show that climate education, when carefully designed and rolled out on a large scale, can increase support for ambitious climate policy. Education is not a substitute for compensatory or regulatory approaches, nor is it sufficient to bridge deep ideological divides. However, it can complement such approaches by strengthening beliefs in the effectiveness of climate policies, creating more constructive engagement with the climate crisis, and, ultimately, increasing the public acceptability of costlier climate policies to mitigate the risks of public backlash. This may lead to a larger coalition in favor of climate mitigation (3). At a time when costly climate policies are increasingly necessary to attain climate targets, and when opposition against climate policy is mounting (39, 41, 42), climate education is an important policy tool for governments in their pursuit of decarbonization.

## Materials and Methods

### Institutional Review Board Approval and Preregistration.

This study was approved by the Ethics Committee at European University Institute on 28 June 2024. All participants provided informed consent prior to participating in each survey. The study was preregistered on OSF on 31 August 2024: https://osf.io/d4vwm.

### Treatment: The 2tonnes Workshop.

The 2tonnes workshop is a 3-h climate education program that teaches participants about climate change and the impacts of individual and collective actions to reduce greenhouse gas emissions. Its name reflects the benchmark set by climate models that average annual emissions must fall to 2tonnes of CO2eq per person to meet the goals agreed under the Paris Agreement. The workshop aims to give a science-based understanding of climate action (*SI Appendix*, section A.1).

In the week prior to the workshop, participants are invited to calculate their personal carbon footprint—covering transport, housing, diet, and consumption of goods and services—using an online tool. Participants receive immediate feedback comparing their footprint to the average French citizen. Those who do not complete this step are assigned a randomly generated footprint for use in the gamified element of the workshop.

The workshop itself is a 3-h session consisting of an introduction, a simulation game, and a debrief. The introduction takes around 30 to 40 min. The facilitator educates participants on topics related to greenhouse gas emissions and carbon footprints, and participants answer quiz-style questions. Participants learn which activities are the most polluting and how their carbon footprint compares to national and workshop averages.

Next, participants take part in the main simulation, which alternates between eight individual and collective decision-making rounds, during which participants are free to decide how to act. The shared objective is to reduce both their individual and collective carbon footprints to below 2tonnes of CO2eq by 2050. Each round iteratively simulates a future point in time where climate conditions continually worsen. Participants choose from a set of actions across domains such as energy, food, housing, transport, industry, and international cooperation. They face a cost constraint in choosing actions. Individual rounds involve private choices about lifestyle, efforts to influence their personal network, and political action. Collective rounds require group deliberation to choose various policy actions, simulating the role of government. After each round, participants see how their choices impact their individual and collective carbon footprint as the model updates. This feature gives them a direct idea about whether their choices were effective or not in reducing their carbon footprint.

Finally, there is a concluding debrief where participants are asked to reflect on their experience, key takeaways, and one action they will implement in their lives going forward. Additional details on the workshop and its content can be found in *SI Appendix*, sections A.1 and A.2.

### Recruitment, Sample Composition, Attrition and Compliance.

We used a snowball sampling procedure to recruit participant universities which already integrate the 2tonnes workshop into their curricula (*SI Appendix*, section B). Ultimately, we recruited 10 universities located across France, in the regions of Paris, Toulouse, Lyon, Nancy, and Clermont-Ferrand. They cover a range of study programs: social sciences and public administration, aerospace engineering, civil aviation, civil engineering and urban planning, chemical and industrial engineering, agronomy and food sciences, veterinary sciences, and business studies.

Across our 10 universities, there were 167 workshops and 1,845 participants. Students in our final sample had an average age of 20, 45% were female, and a majority had at least one parent earning above the national average income. Full summary statistics are reported in *SI Appendix*, Table S2. The randomization procedure is presented in Fig. 1*B*. *SI Appendix*, section C includes information on covariate balances between treatment and control (*SI Appendix*, Table S6). As demonstrated by power calculations in *SI Appendix*, section E.2 (*SI Appendix*, Table S13), our main analysis is adequately powered: required N = 1,544 to 1,702 for 80% power, actual N = 1,845 (achieved power ≈ 0.83 to 0.86).

We collaborated with administrative staff at each university to coordinate randomization procedures and distribution of online surveys. The mandatory nature of the workshop helped to mitigate against self-selection bias. The overall workshop-day attrition rate—which includes both absences and participation without a completed survey—1 was 19% (*SI Appendix*, section B and Table S3). This rate differed between treatment (22%) and control (16%) groups. The higher rate for the treatment group likely reflects the lengthy nature of the workshop. Participation without completion of the survey was 13%, though this was lower for more experienced workshop facilitators. *SI Appendix*, Table S6 shows that there was no significant imbalance between control and treatment arms across 15 variables plausibly unaffected by treatment (F-test = 1.05), indicating that attrition rates did not differ substantially between groups. Empirically, we also implement bounding analyses following (43), which confirm that our main conclusions are robust to attrition. Further details on workshop attrition can be found in the *SI Appendix*, section B.

Of the 1,845 workshop participants we surveyed, 330 opted in to complete an endline survey 6-wk later. As a result, our persistence effects must be interpreted with caution. *SI Appendix*, Table S7 presents balance tests which show that participants who took the endline survey were more supportive of costly climate policies at baseline, identified as more left-wing, and exhibited lower carbon-intensive lifestyles (less flying, less meat). Importantly, however, treatment status was balanced.

Overall, we cannot claim that our endline sample is representative of the baseline sample and thus limits the generalizability of the persistence effects documented in Fig. 4. We interpret these endline results as suggestive evidence of the medium-term effect of the workshop on policy preferences, albeit for a subset of our total sample.

### Measurement of Outcome Variables.

We collected data on support for three costly climate policies. Each respondent was asked to indicate their support on a 5-point Likert scale. We then created a binary indicator for policy support, coded as 1 if the respondents selected “support” or “strongly support” on the scale, and 0 if they show indifference or opposition to the policy. For our first two policy outcomes—a tax on beef products and a ban on flights—we provided respondents with the following descriptions and then asked their support:

- *Imagine that, to fight climate change, the government decides to limit the consumption of beef. A high tax on beef is put in place, doubling its price.*
- *Imagine that, to fight climate change, the government decides to limit the use of aeroplanes. A ban on national and international flights for destinations accessible by train or bus within 6 h is put in place.*

Then for our third policy outcome—a ban on meat in the school canteen—we asked respondents the following question:

- *Would you be in favor of introducing exclusively vegetarian menus in the university canteen?*

For our behavioral outcome, we first offered participants an option to enter a lottery with a €100 prize (conditional on also completing a follow-up survey 6 wk later). If they opted in, they are then given the opportunity to donate a portion of their potential winnings to Réseau Action Climat, an environmental NGO which does advocacy work on the topics of the policies we ask about earlier in the survey. Donation allocations were elicited using a sliding scale, with €1 increments ranging from €0 to €100.

Additional information on the measurement of other variables used in our analysis is in *SI Appendix*, section D. The entire questionnaire can be found in *SI Appendix*, section I.

### Estimation Strategy.

The randomization strategy allows us to identify the immediate effect of the workshop on climate policy preferences. Our main estimand of interest is a finite population estimand at the student level. More specifically, we are interested in the average treatment effect of the workshop on our target population of students. We use the following estimation strategy:

[1] $$\begin{array}{c} Y_{\mathrm{ijsu}}=\beta_{0}+\beta_{1}Z_{j}+\delta_{s}+\theta_{u}+\epsilon_{\mathrm{ijsu}}, \end{array}$$

where *i* is the individual in the workshop *j* in experimental block *s* at university *u*, *Z* is a binary treatment indicator, and *Y* is the outcome of interest. The specification includes university fixed effects ($\theta_{u}$) and experimental block fixed effects ($\delta_{s}$) and uses CR2 cluster-robust SE at the workshop to account for the collective nature of the treatment. The coefficient $\beta_{1}$ captures the average treatment effect.

To test the persistence effects 6 wk after the workshop, we use two empirical strategies. First, using a within-group design, we estimate the following:

[2] $$\begin{array}{c} Y_{\mathrm{it}}=\beta_{0}+\beta_{1}T+\theta_{i}+\delta_{w}+\epsilon_{\mathrm{it}}, \end{array}$$

where *i* is the respondent, *T* is a dummy indicating whether an individual’s response was recorded at time of the workshop or 6 wk later, and *Y* is the outcome of interest. We use an individual fixed effect (*θ*), a workshop fixed effect ($\alpha_{w}$) and cluster-robust SE CR2 at the individual level. $\beta_{1}$ is the average treatment effect we are interested in.

Next, using a between-group design, we estimate a difference-in-differences specification that interacts treatment status and survey wave (workshop day vs. endline). This approach compares changes in support for costly climate policies between treated and control respondents over time. The specification is

[3] $$\begin{array}{c} Y_{\mathrm{it}}=\beta_{0}+\beta_{1}Z_{i}+\beta_{2}Post_{t}+\beta_{3}\left(Z_{i}\times Post_{t}\right)+\theta_{u}+\epsilon_{\mathrm{it}}, \end{array}$$

where $Z_{i}$ is a treatment indicator, $Post_{t}$ is a posttreatment dummy (equal to 1 for endline responses), and $\theta_{u}$ are university fixed effects. The coefficient of interest is $\beta_{3}$, which captures the persistence of treatment effects relative to the control group. SE are clustered at the workshop level.

Across all specifications, our primary estimand is the ITT effect, in line with our preanalysis plan. For robustness, we also estimate LATE, conduct leave-one-university-out sensitivity checks, and examine potential confounding by time-of-day of the workshop. Further implementation details, as well as additional robustness analyses, are reported in *SI Appendix*, section F.

## Supplementary Material

Appendix 01 (PDF)

## Data Availability

Anonymized data (survey data) and replication materials are available on Harvard Dataverse at (https://dataverse.harvard.edu/dataset.xhtml?persistentId=doi:10.7910/DVN/TPWXG8) (44).
