# Supplementary material for: Educational policies can strengthen climate coalitions
Source: Proc Natl Acad Sci U S A. 2026 May 14;123(20):e2533821123. doi: 10.1073/pnas.2533821123 (PMC13187721; doi:10.1073/pnas.2533821123)
Supplement: Supplementary file 1 [file pnas.2533821123.sapp.pdf]

Supporting Information for "Educational policies can strengthen climate coalitions"

April 28, 2026

Contents

A The 2tonnes Workshop **1**

B Descriptive Statistics, Recruitment Strategy, Compliance and Attrition **7**

C Randomization and Covariate Balance **12**

D Measurement **14**

E Further Results **19**

F Robustness Checks **47**

G Comparison of Effect Sizes **55**

H Additional Experimental Elements **57**

I Full Questionnaire **61**

A The 2tonnes Workshop

A.1 Origins and methodology of the 2tonnes workshop

In recent years, French NGOs have developed standardized climate change workshops that educate participants, both within and beyond formal education settings, on the science of climate change and the transformations required to reduce greenhouse gas emissions in accordance with the targets of the Paris Agreement. Since their beginning in 2018, more than two million people have participated in the “Climate Fresk”, which aims to educate participants about the causes and effects of climate change. The “2tonnes” workshop, studied in this article, was launched in 2020, with over 260,000 participants. This workshop takes a more policy- and action-oriented approach to the climate crisis, focusing on individual- and societal-level changes needed to transition to a low-carbon society where individual carbon footprints are reduced to 2tonnes of CO2 equivalent per year by 2050.

The 2tonnes workshops are deployed jointly by two organizations. One organization is a non-profit association responsible for running free workshops for a large and not predefined audience (“the general public”). The other is a social economy start-up (i.e. simplified joint stock company) which organizes workshops in professional settings, i.e. for companies and public authorities.[1] The theory of change behind the 2tonnes workshops centers on the idea that learning about the problem and its solutions can encourage people to change both the way they think about the climate crisis and how they act to address it.

The workshop relies on a simulation of low-carbon transition scenarios and carbon footprints based on participants' decisions. Prior to the workshop, participants are asked to calculate their individual carbon footprint. Following an introductory session (see SI Section A.2), the workshop consists of eight decision-making rounds that alternate between individual-level and collective-level actions. In each round, participants select action cards designed to reduce their personal or collective carbon footprint. At the end of each round, the simulation tool updates the footprint based on these choices, allowing participants to learn about the effectiveness of their decisions for climate change mitigation and to compare their results with those of their peers.

The calculation methodology is based on three interacting models.[2] First, the "carbon footprint model" uses data from organizations such as ADEME (the French Agency for the Ecological Transition), the OECD, and the FAO. The "action model" updates the data of participants and the population, based on assumptions from well-known transition scenarios in France (RTE, négaWatt, ADEME.). The "influence model" quantifies the societal dynamics taken into account in the simulation, drawing on concepts from behavioral sciences. Therefore, the workshop aims to give a science-based understanding of climate action, and participants are free to decide how to act during the simulation.

[1]See: https://www.2tonnes.org/a-propos

[2]See: https://www.2tonnes.org/modele-sources-donnees

A.2 Workshop content

This section gives details on the main questions and points covered during the three-hour 2tonnes workshop.

Part 1: Introduction

The first part of the workshop is an introduction to key facts about climate change. It is a way to ensure all participants know the key concepts used during the workshop and to "break the ice" between participants. The facilitator typically asks the following questions:

1. How have poverty rates changed over time?
2. What is the average life expectancy?
3. How much have global temperatures risen since the last glacial period?
4. Are humans responsible for this?
5. What is the Paris Agreement goal? And how does this relate to the concept of “2tonnes”?
6. What are greenhouse gases?
7. What is a carbon footprint?
8. Which are the greatest sources of emissions amongst French citizens?
9. How do carbon footprints vary across countries?
10. How does your footprint compare to the average French citizen? And to others in the workshop group?

Part 2: Simulation Game

The simulation game follows the introduction. We summarize below the main learning points from each of the eight decision-making rounds of the game. The points can vary according to the group composition and topics of discussion of each workshop group. The points summarized here are those given most prominence on the slide deck, thus representing the most likely learning points.

- Round 1: Reduction in red meat consumption has by far the largest impact of any action that can be taken regarding individual food consumption habits.
- Round 2: changing agricultural practices (for example, using less fertilizer) and switching to biogas instead of "natural" gas are impactful policies.

- Round 3: Travelling alone in a combustion engine car is highly emitting, and can reach levels comparable with flying. Car-sharing reduces this. Reducing or stopping flying, investing in an electric vehicle, using public transport and travelling less are effective ways to reduce your carbon footprint.
- Round 4: Investment in vehicle technologies and public transport is an effective way to reduce emissions.
- Round 5: Investing in a heat pump or insulation in your home is an effective way to reduce your carbon footprint. Changing to green electricity can also be beneficial, although in countries such as France, the energy mix is already low-carbon, meaning there are fewer gains to be made here.
- Round 6: Optimizing energy in the tertiary sector, investing in the circular economy (using fewer raw materials) and supporting other countries in their energy transitions are beneficial ways to reduce carbon emissions.
- Round 7: If you have not yet become vegan and stopped flying, these are some of the most impactful actions you can take.
- Round 8: The group must choose between developing renewable energies and renewing investment in nuclear power (or opt for both). Radical policies such as import bans on products from high-emitting countries or carbon quotas on individuals are discussed. Participants learn that radical measures can be very effective, but will debate how desirable or feasible such policies are.

Part 3: Debrief

Below are the key points covered during the workshop debrief:

1. Whether the goal of an average of 2tonnes per person is reached depends on decisions made by the participants during the game.
2. Reaching the goal of 2tonnes is an ambitious target. The model used in the game is a simplification of reality, but it shows that significant reductions in greenhouse gas emissions are possible.
3. Action should be taken in all sectors, both at the individual and collective levels.
4. Citizens, governments and firms must all act to create change.
5. The facilitator presents a recap of actions that can be taken to reduce greenhouse gas emissions in the topics covered in the workshop.

6. The group are invited to share reflections on how they feel after taking the workshop, one key piece of information they have learned, and one action they want to commit to moving forward.

7. The workshop concludes with the message that reaching the goal of 2tonnes is ambitious, but necessary. It is reaffirmed that there are many ways to reach this goal, and the energy transition also presents opportunities.

8. The workshop ends with information about how participants can themselves become a volunteer facilitator.

A.3 Cost effectiveness of the 2tonnes workshop

This section examines the cost-effectiveness of the 2tonnes climate education workshop compared to other educational interventions designed to increase support for climate policies. The 2tonnes workshop is a hybrid intervention: it combines the deliberative, participatory features of a workshop with an interactive simulation that models policy impacts across time. This makes it directly comparable to workshop-based educational programs and online simulation-based interventions.

Consequently, we compare the cost-effectiveness of this hybrid workshop-and-simulation intervention to (i) traditional workshop-based stand-alone courses and (ii) online simulation tools. We define an intervention's cost-effectiveness as the estimated effect size divided by the implementation cost per participant. Because other studies do not explicitly measure policy support, we approximate their policy support effects using a bounding exercise. This allows us to compare the cost-effectiveness of the 2tonnes workshop with other interventions in increasing policy support for climate policy.

Table 1 summarizes the results. We estimate that the 2tonnes workshop is more cost-effective than both stand-alone workshop courses and online simulation games in increasing support for costly climate policies.

Table 1. Cost-effectiveness of climate education and engagement interventions

**Intervention**	**Outcome**	**Effect size (pp)**	**Cost/participant (€)**	**Cost-effectiveness (€/pp)**
2tonnes workshop	Policy support	7	30	4.3
Stand-alone climate workshops	Policy support	12	100	8.3
Online simulation (En-ROADS, World Climate)	Policy support	2	12	6.0

Notes: Cost-effectiveness is calculated as the cost per percentage point change in the relevant outcome variable. Effect sizes for the *2tonnes* workshop are derived from the main results shown in Figure 2 in the main paper, while other estimates are based on benchmarks derived from the literature [1, 2, 3] All costs are expressed in euros (excl. tax) per participant.

Costing the 2tonnes Workshop

In university settings, the 2tonnes workshop can be implemented in a modular format at a cost ranging from €15 to €30 per student, depending on whether facilitation is provided internally by faculty or externally by trained facilitators. In the lower-cost scenario, the university provides its own facilitator. Given a three-hour session, a facilitator rate of €60 per hour, and a group size of 15 participants, the facilitation cost is approximately:

$$\frac{60 \times 3}{15} = 12 \text{ € per person.}$$

Including all materials and logistics, we assume an overall cost of €30 per participant.

Cost-effectiveness is computed as:

$$\text{Cost per pp change} = \frac{\text{Cost per participant}}{\text{Effect size (in pp)}}.$$

We find that with an effect size of 7 percentage points and a cost of €30 per participant, the cost-effectiveness ratio of the 2tonnes workshops' effect on policy support is €4.3 per percentage point.

Stand-alone Climate Workshops

We first compare the cost-effectiveness of the 2tonnes workshop with existing climate stand-alone workshops. Specifically, we examine short, stand-alone climate-related courses or certificate programs, typically offered through continuing education or executive training. Such programs entail instructor fees, coordination, digital platform access, and materials. For instance, the *Carbon Literacy Training – Carbon Charter* program consists of three 2-hour sessions and costs approximately €100 per participant[3]. These stand-alone courses are workshop-based but do not incorporate an interactive simulation. In this sense, they isolate only one component of what the 2tonnes workshop provides.

In order to construct a benchmark effect of other stand-alone courses on climate change, we rely on a comprehensive meta-analysis of climate education interventions and their effects on a range of outcomes from Aeschbach et al (2025) [1]. To calculate the cost-effectiveness of other stand-alone courses on climate change, we compute Hedges' g values from Aeschbach et al (2025) [1] of 0.29 for policy support. We also assume a baseline support level of 50%. This allows us to approximate the effect in percentage points as:

$$\Delta p \approx g \times \phi(\Phi^{-1}(0.5)) \approx g \times 0.3989.$$

[3] `https://carboncharter.org/events/carbon-literacy-training-3/`

This yields effects of approximately 12 pp (for $g = 0.29$). The corresponding cost-effectiveness ratio is therefore:

$$100/12 \approx 8.3 \text{ € /pp.}$$

This analysis indicates that other stand-alone climate education workshops are twice as costly as the 2tonnes workshop in building support for costly climate policies. This implies that the hybrid 2tonnes model achieves comparable or larger impacts on policy-related outcomes at substantially lower cost per participant.

Online simulation tools on climate change

Simulation-based educational tools, such as the *World Climate* and *En-ROADS Climate Action Simulation*, are freely available and primarily incur facilitation costs. These workshops have reached over 468,000 participants in 181 countries, notably within universities [2]. Evaluations report substantial gains in knowledge and significant improvements in perceived policy effectiveness [3]. However, these interventions rely solely on interactive simulation and do not include the facilitated small-group deliberation that characterizes the 2tonnes workshop.

Using a 2 pp (for $g = 0.05$) increase in policy support [2, 3] and a facilitation cost of €12 per participant, we obtain:

$$12/2 = 6 \text{ €/pp.}$$

This analysis indicates that the 2tonnes workshop is again a more cost-effective approach to increasing support for costly climate policies. Compared to online simulation tools, the 2tonnes workshop generates substantially larger shifts in policy support at a comparable or lower cost-per-point, again reflecting the combined strengths of the workshop-plus-simulation format.

Taken together, these comparisons show that the 2tonnes workshop – by combining the interactive elements of simulation with the collective, deliberative features of a workshop – is a more cost-effective intervention than either stand-alone format. The hybrid design, therefore, appears to deliver disproportionately large gains relative to its modest implementation cost.

B Descriptive Statistics, Recruitment Strategy, Compliance and Attrition

Table 2 shows the summary statistics of key variables across the students we surveyed. The average age of the students was 20, and 45% of respondents were female. A majority of students had at least one parent earning above the national average income. Compared to the general French public, our study population is much younger, higher educated, and considerably more left-wing.

Table 2. Descriptive statistics of the student sample

Variable	N	Mean	Min.	Q1	Q2	Q3	Max.
Socio-demographic characteristics							
Age	1830	19.90	17.00	19.00	20.00	21.00	27.00
Female (%)	1828	0.45	0.00	0.00	0.00	1.00	1.00
Highest parent's income (in €)	1432	4368.02	1550.00	2050.00	4150.00	7500.00	7500.00
Childhood in rural area (%)	1832	0.41	0.00	0.00	0.50	1.00	1.00
Enjoyed the workshop (%)	919	0.81	0.00	0.70	0.80	1.00	1.00
Party identification							
Ecological (%)	1845	0.07	0.00	0.00	0.00	0.00	1.00
Left (%)	1845	0.13	0.00	0.00	0.00	0.00	1.00
Center-left (%)	1845	0.17	0.00	0.00	0.00	0.00	1.00
Center (%)	1845	0.08	0.00	0.00	0.00	0.00	1.00
Center-right (%)	1845	0.10	0.00	0.00	0.00	0.00	1.00
(Far-)Right (%)	1845	0.05	0.00	0.00	0.00	0.00	1.00
Survey quality indicators							
Finished the survey (%)	1845	0.99	0.00	1.00	1.00	1.00	1.00
Length of the survey (min)	1845	16.09	1.48	5.75	6.97	8.40	7484.18
Outcomes: support for costly climate policies							
Flight ban (%)	1810	0.59	0.00	0.00	1.00	1.00	1.00
Beef tax (%)	1800	0.38	0.00	0.00	0.00	1.00	1.00
Meat-free canteen (%)	1818	0.40	0.00	0.00	0.00	1.00	1.00
Mediator: perceived effectiveness							
Flight ban (%)	1809	0.72	0.00	0.00	1.00	1.00	1.00
Beef tax (%)	1804	0.53	0.00	0.00	1.00	1.00	1.00

Note: Socio-demographic characteristics, survey quality indicators, outcomes and moderators variables for the studied sample. Column 2 reports the number of non-missing observations (N). Columns 3 to 8 show the mean, minimum, first quartile (Q1), median (Q2), third quartile (Q3), and maximum values. Childhood in rural area = whether respondent spent majority of years 10-18 in a rural area, where 1=rural, 0.5=peri-urban, 0=urban. Party ID follows Cagé and Pikkety's 2025[4] classification.

The ten universities in our sample were recruited in several steps. First, we established contact with the 2tonnes association, which directed us to several universities that were organizing 2tonnes workshops as part of their curricula. We then reached out to other universities thanks to the recommendations of our established contacts. Beyond snowballing, we sought to include in our sample a diversity of geographical locations within France (region of Paris, Toulouse, Lyon, Nancy, and Clermont-Ferrand) and of fields of study (social sciences and public administration, aerospace en-

gineering, civil aviation, civil engineering and urban planning, chemical and industrial engineering, agronomy and food sciences, veterinary sciences and business studies).

Table 3 presents descriptive statistics on the number of workshops evaluated and of students in each participating university. The initial number of students refers to the number of students that administrative staff expected to participate in the workshops. This data was provided to us in advance of the workshops during the randomization phase. The number of students reached is the number of students who completed the survey (either before the workshop for workshops assigned to the control condition, or after the workshop for workshops assigned to the treatment condition). We surveyed 1,845 of the 2,274 students we expected to be included in the workshops, implying an overall attrition rate of 19%. Within this overall attrition rate, two types of attrition can be observed. Pre-attendance attrition refers to cases where students did not attend the workshop, whereas post-attendance attrition refers to students who did participate in the workshop but did not fill out our survey.

Table 3. Descriptive statistics of number of workshops, compliance and attrition for the full sample and by university

	**Workshops**	**Students**		**Compliance (%)**	**Overall attrition (%)**		
		Initial	Reached		All	Control	Treated
Full sample	167	2274	1845	93	19	16	22
UNI1	12	153	130	100	15	20	10
UNI2	19	213	197	100	8	1	13
UNI3	16	277	168	100	39	32	47
UNI4	17	190	154	95	19	17	21
UNI5	14	221	192	96	13	12	14
UNI6	21	277	189	68	32	29	34
UNI7	10	121	94	85	22	20	25
UNI8	2	20	17	100	15	0	30
UNI9	4	50	35	100	30	33	27
UNI10	41	581	508	94	13	9	16
UNI11	11	171	161	100	6	3	9

Note: Compliance refers to the proportion of participants in the treatment condition to which they were assigned. Overall attrition is composed of both pre-attendance attrition (i.e. absence) and post-attendance attrition (i.e. non-completion of the survey). We conduct our study in ten universities, but report eleven in this table, since UNI11 is the same institution as UNI5, but a different student cohort.

We achieved a compliance rate of of 93%, meaning that 93% of study participants were in the treatment condition to which we assigned them. A large part of the non-compliance we observe comes from two universities where, due to last-minute administrative changes or confusion, entire workshops switched treatment assignment. We report the Intent-to-Treat (ITT) effect in the main results and report the Local Average Treatment Effect (LATE) in the Appendix F.3 as a robustness check.

To assess pre-attendance attrition, we rely on facilitator reports, which were collected from 111

of the 167 workshops. On average, pre-attendance attrition was approximately 8.5%, with similar rates for treated (8.21%) and control workshops (8.7%). Since workshop attendance was mandatory across all participating universities, pre-attendance attrition remained relatively low. However, it was not zero, as students may have been unable (e.g., due to illness) or unmotivated to attend. Because students were unaware of their treatment status until they arrived at the workshop, pre-attendance attrition is unlikely to differ systematically between treatment and control groups. As a result, this type of attrition does not compromise our ability to identify the Average Treatment Effect[5].

The more concerning issue is post-attendance attrition, as this may violate the core assumption required to identify the causal effect of treatment: specifically, that treatment assignment is independent of potential outcomes. We estimate that post-treatment attrition accounts for 10.5 percentage points of the overall 19% attrition rate.

In the absence of complete data for pre-attendance and post-attendance attrition, we employ strategies to further assess overall attrition which help to evaluate any potential threat to the validity of our inferences. First, we assess reasons for non-attendance. In five universities where students were assigned to workshops (covering 600 students), we examine whether non-attendees differ from attendees. Table 4 presents the results of this analysis. Gender was the only covariate consistently recorded across all four universities, and we find no systematic differences between attendees and non-attendees. For one university (approximately 250 students), we have further background information on non-participants. Notably, students of an environment-related topic (biology) were significantly less likely to be absent. They constituted 26% of survey respondents but only 6% of non-participants, a difference statistically significant at the 0.1% level. This suggests that students less interested in environmental topics were more likely to disengage from the workshop and survey, despite the mandatory nature of the workshop.

Table 4. Pre-treatment covariates balance between workshop attendees and non-attendees

	**Average**		**diff**	**p-value**
	Absent	Attended		
Female	0.345	0.375	0.0295	0.45
Biology bachelor	0.0625	0.259	0.197	0.001
Maths bachelor	0.312	0.187	-0.126	0.16
Physics bachelor	0.0938	0.119	0.0254	0.66
Computer science bachelor	0.0938	0.171	0.0772	0.196
Civil servant students	0.531	0.565	0.0335	0.73
Prep school students	0.574	0.626	0.0523	0.539

Note: The table represents the average of pre-treatment covariates for students who did and did not attend the workshop across four universities. The third column shows the difference between the two groups and the p-value from a t-test is shown in the fourth column. The female variable is present in all four universities. The curricula variables are for one university only.

Second, we examine whether facilitator demographics predict overall attrition rates. We calculate the number of missing responses per workshop (as self-reported by facilitators) and regress it on key facilitator characteristics. Facilitators without prior workshop experience exhibit significantly higher attrition—an increase of 19 percentage points—compared to experienced facilitators. This suggests that first-time facilitators may have struggled to balance the demands of leading a workshop while adhering to the evaluation protocol, likely contributing to the observed attrition.

Third, we assess whether attrition rates differ between the treatment and control groups. Descriptively, the combined pre- and post-attendance attrition rate is 22% in the treatment group and 16% in the control group. We use Welch's two-sample t-test to evaluate whether this difference is statistically significant. As reported in column 2 of Table 5, the 6-percentage-point difference—equivalent to approximately one student in a workshop of 15—is significant at the 5% level. The slightly higher attrition among treated units may reflect challenges in sustaining student engagement throughout the session. In anticipation for such unbalances, we instructed facilitators to administer the post-survey before the final segment of the workshop. The covariate balance table in the next section text shows no significant differences between groups, suggesting that attrition does not systematically bias the comparison between treatment and control units.

Table 5. Statistical association between facilitator demographic characteristics and attrition rates

	**Attrition rates**				
	(1)	(2)	(3)	(4)	(5)
Constant	15.52^{***}		2.15		
	(1.94)		(8.33)		
Treatment	$6.03^{\cdot}$	5.91^{*}	3.38	4.10	4.59
	(3.14)	(2.82)	(3.62)	(3.25)	(3.43)
Treatment × No experience					−3.25
					(11.98)
Female			3.43	5.86	5.82
			(3.53)	(3.73)	(3.80)
Age			0.23	0.19	0.19
			(0.17)	(0.16)	(0.16)
No experience			19.03^{**}	8.96	10.55
			(6.66)	(6.34)	(7.38)
Experience			−0.01	−0.09	−0.09
			(0.08)	(0.08)	(0.08)
University FE	No	Yes	No	Yes	Yes
Adj. R^2	0.02	0.21	0.07	0.27	0.27
Num. obs.	167	167	112	112	112

Note: The table represents the result of the OLS regression between facilitator key demographic characteristics and attrition rates. Variables included are the treatment status of the workshop, gender, and age of the facilitator, a dummy for whether the facilitator have at least one experience in facilitating the workshop, and a variable for the number of workshop facilitating. In parenthesis robust standard errors. $^{***}p < 0.001$; $^{**}p < 0.01$; $^{*}p < 0.05$; $^{\cdot}p < 0.1$

The gold standard for addressing attrition, as discussed by Coppock et al. (2017) [6], is the double-sampling strategy. However, in the absence of baseline data, we are unable to follow up with non-respondents. Instead, we implement the bounding approach proposed by Lo, Renshon, and Bassan-Nygate (2024) [5]. Figure 1 presents the results. Under sharp bounds, we assign the highest possible outcome to control units with missing data and the lowest possible outcome to treated units with missing data. This reflects an extreme scenario in which only the most pro-climate policy students in the control group and the least pro-climate policy students in the treatment group fail to respond. Under this assumption, the Average Treatment Effect (ATE) for the university student population is undefined. However, this scenario is unlikely to be realistic. Under milder bounds—where the probability of receiving the highest score is set at 0.67 for missing control observations and 0.33 for missing treated observations—we find that the ATE is likely to be positive.

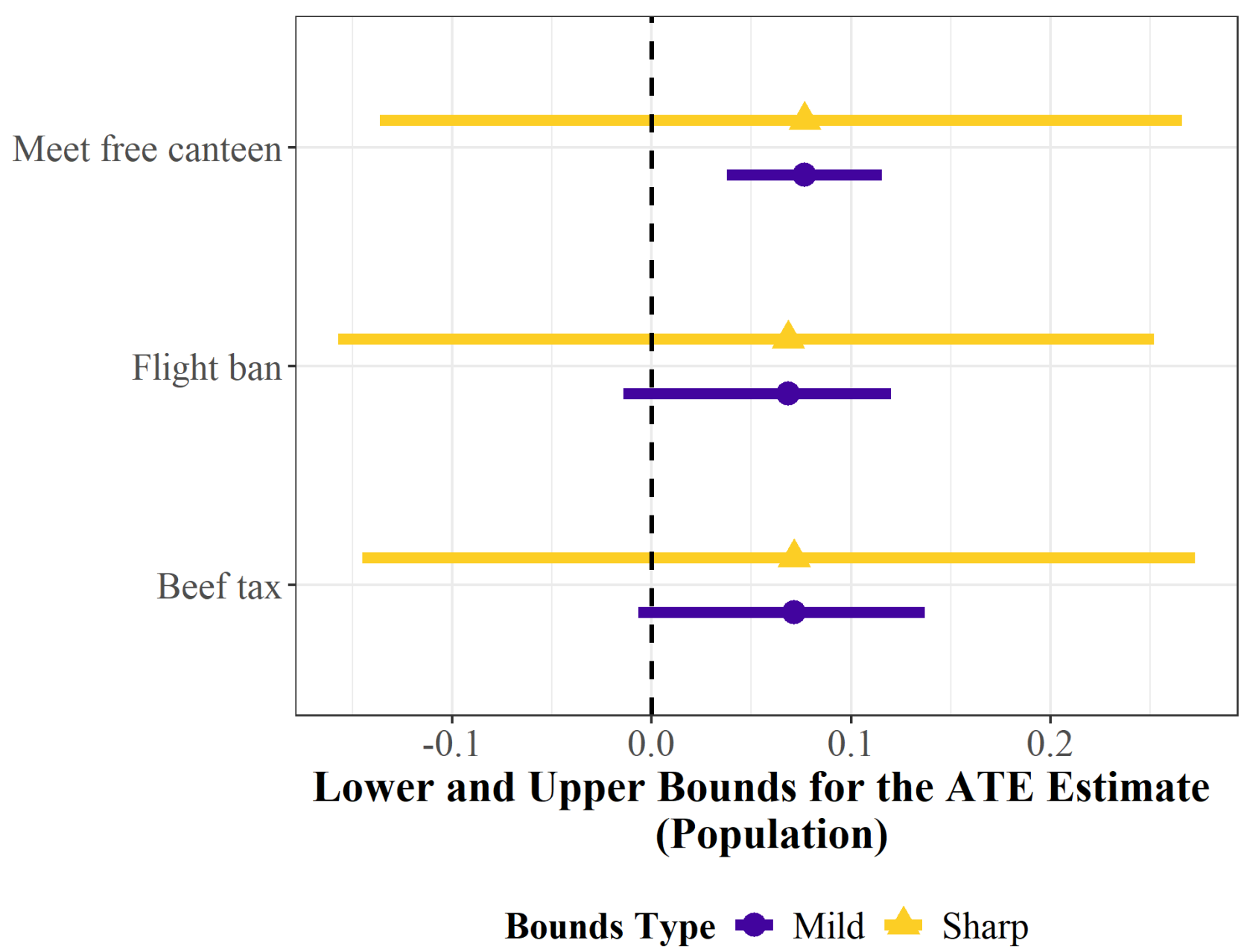

Figure 1. Sharp and mild bounds estimation of the Average Treatment Effect of participation into the 2tonnes workshop for the population of university students

C Randomization and Covariate Balance

Table 6. Covariate balances for covariates expected to not be impacted by the workshop

	Control (C)	Treatment (T)	(C) - (T)
Progress	99.74	99.77	0.03
Survey finished	0.99	0.99	0.00
Length	1387.38	578.97	-808.42
Age	19.88	19.84	-0.03
Female	0.45	0.45	0.00
Income	4334.90	4333.97	-0.92
Childhood in rural area	0.39	0.41	0.02
Participation in Climate Fresk	0.42	0.42	-0.00
Carbon footprint measured	0.80	0.77	-0.03
Demonstrated for non-environmental cause	0.07	0.05	-0.01
Demonstrated for environmental cause	0.02	0.01	-0.01
Donation to a climate NGO	0.06	0.07	0.00
Shared information on climate change	0.18	0.20	0.02
Flight consumption (log)	0.20	0.21	0.01
Meat consumption	2.48	2.53	0.05
**F-test: 1.05**			

Note: Income = income of the highest-earning parent (multiple choice within income brackets). Childhood in a rural area = whether respondent spent majority of years 10–18 in a rural area, where 1 = rural, 0.5 = peri-urban, 0 = urban. Participation in Climate Fresk = binary variable, prior participation in a similar climate workshop. Carbon footprint = whether participant calculated carbon footprint before the workshop. Flight consumption (log) = logarithmic transformation of number of flights taken in Europe within past year. Meat consumption = times per week eating red meat. $^{***}p < 0.001$; $^{**}p < 0.01$; $^{*}p < 0.05$; $^{+}p < 0.1$.

Table 7. Covariate balance between participants who took only the midline survey and those who completed both midline and endline

	**Midline Only**	**Midline & Endline**	**Difference**
Workshop treatment status	0.49	0.50	0.01
Age	19.83	20.23	0.40***
Female	0.45	0.49	0.04
Income	4492.91	3858.69	-634.22***
Participation in Climate Fresk	0.42	0.48	0.06+
Carbon footprint	0.77	0.84	0.07**
Political activism index	0.32	0.35	0.03
Donation to climate NGO	0.07	0.05	-0.02
Sharing climate info	0.18	0.21	0.03
Environmental strike participation	0.01	0.02	0.01
Any strike participation	0.06	0.07	0.01
Flight consumption (log)	0.22	0.16	-0.06***
Meat consumption	2.52	2.38	-0.14*
Support for beef tax	2.79	3.04	0.25**
Support for flight ban	3.36	3.80	0.44***
Support for meat-free canteen	2.79	3.16	0.37***
Perceived effectiveness - beef	3.24	3.43	0.19**
Perceived effectiveness - flight	3.76	4.04	0.28***
Party ID: left	0.12	0.16	0.04
Party ID: center-left	0.15	0.28	0.13***
Party ID: center	0.08	0.07	-0.01
Party ID: center-right	0.10	0.08	-0.02
Party ID: (far-)right	0.05	0.05	0.00
Conservatism index	0.15	0.13	-0.02
**N**	1516	330	

Note: The table displays mean values for a series of variables. These variables are all measured at the initial survey. The aim here is to compare differences between those who opted-in to take the endline survey and those who did not. The difference column represents the results of a series of t-tests. Income = income of the highest-earning parent (multiple choice within income brackets). Participation in Climate Fresk = binary variable, prior participation in a similar climate workshop. Carbon footprint = whether participant calculated carbon footprint before the workshop. Flight consumption (log) = logarithmic transformation of number of flights taken in Europe within past year. Meat consumption = times per week eating red meat. Party ID refers to the political party to which the respondent feels closest, using Piketty and Cagé's 2025 ([4]) classification. Conservatism index = center-right or right. $^{***}p < 0.001$; $^{**}p < 0.01$; $^{*}p < 0.05$; $^{+}p < 0.1$.

D Measurement

The full questionnaire for our survey is provided in Section I below. Here, we provide information on the measurement of our costly climate policy outcome variables and behavioral outcomes. We also describe how we measured our mechanisms and manipulation checks, i.e., policy effectiveness, knowledge, emotions, and psychological distance. Finally, we describe how we operationalized our moderators.

D.1 Measurement of Outcome Variables

We collected data on support for three costly climate policies. Each policy was chosen due to its stringent nature, i.e., it would impose visible costs on individuals. Additionally, we selected policies which are to some degree salient in the public debate in France.

Each respondent was asked to indicate their support on a 5-point Likert scale. We then created a binary indicator for policy support, coded as 1 if the respondents selected "support" or "strongly support" on the scale, and 0 if the show indifference or opposition to the policy. For our first two policy outcomes – a tax on beef products and a ban on flights – we provided respondents with the following descriptions and then asked their support:

- *"Imagine that, to fight climate change, the government decides to limit the consumption of beef. A high tax on beef is put in place, doubling its price."*
- *"Imagine that, to fight climate change, the government decides to limit the use of aeroplanes. A ban on national and international flights for destinations accessible by train or bus within 6 hours is put in place."*

Then for our third policy outcome – a ban on meat in the school canteen – we asked respondents the following question:

- *"Would you be in favor of introducing exclusively vegetarian menus in the university canteen?"*

For our behavioral outcome, we first offered participants an option to enter a lottery with a €100 prize (conditional on also completing a follow-up survey six weeks later). If they opted in, they are then given the opportunity to donate a portion of their potential winnings to Réseau Action Climat, an environmental NGO which does advocacy work on the topics of the policies we ask about earlier in the survey. Donation allocations were elicited using a sliding scale, with €1 increments ranging from €0 to €100.

D.2 Measurement of Mechanisms and Manipulation Checks

Our two pre-registered mechanisms hypothesized that an increase in support for costly climate among participants would be driven by a) a decrease in psychological distance to the climate crisis, and b) an increase in belief in policy effectiveness.

Psychological Distance

We measure psychological distance with the following survey question:

- *"To what extent do you feel that climate change will affect your life personally in the next 10 years?"*
  - Measured on a 5-point Likert scale: 1 (Not at all) – 5 (Extremely)

When operationalizing this variable for analysis, we standardize the values so that the values run from 0 to 1. We also note here that this variable does not adequately capture the multi-faceted nature of the concept of psychological distance. In practice it captures the longer-term element of the concept, which the workshop may be less likely to activate compared with other elements. Additionally, this measure can be thought of as the perceived personal impact of climate change.

Effectiveness Beliefs

We measure respondents' effectiveness beliefs for two of our three costly climate policies – the beef tax and the flight ban. Specifically, we ask respondents the following survey questions:

- *"A high tax on beef products would be an effective means to combat climate change"*
- *"A ban on national and international flights for destinations accessible by train or bus within 6 hours would be an effective means to combat climate change"*
  - Both measured on a 5-point Likert scale: 1 (Strongly Disagree) – 5 (Strongly Agree)

As with our outcome variables, we created binary indicators for policy effectiveness beliefs, coded as 1 if the respondents selected "agree" or "strongly agree" on the scale, and 0 if they showed indifference or disagreement. These binary variables are what we use in the analyses presented in the main text.

Knowledge

We measured respondents' knowledge using two multiple-choice survey questions which both have one correct answer:

1st Knowledge Question:

- Which of the following contributes the most to the carbon footprint of the average French citizen?
  - Car Usage
  - Meat Consumption
  - Airplane Travel
  - Heating
  - Use of digital and online services
  - I don't know
- The correct answer is "Car usage". Thus, measurement will be a binary variable which takes the value of 1 ("Car usage") and 0 (all other answers).

2nd Knowledge Question:

- What is the estimated contribution of beef consumption to the carbon footprint of the average French person?
  - 3%
  - 15%
  - 30%
  - I don't know
- The correct answer is "15%". Thus, the measurement will be a binary variable which takes the value of 1 (15%) or 0 (all other answers)

We operationalize knowledge empirically by first constructing two binary variables indicate whether respondents correctly (1) or incorrect (0) answered each question. We then take the average of these two variables to construct one combined knowledge variable which we use in our regression analyses.

In the endline survey, we only asked the first of these questions. Our endline measure for knowledge is thus based only on answers to this one question.

Emotions

We measure emotions using the following survey question:

- When you think about climate change, what is your reaction? (Please choose up to 2 options).
  - Motivated
  - Anxious

- Optimistic
- Concerned
- Indifferent
- Calm
- Hopeful
- Guilty
- Sad
- Angry
- None of these emotions
- I don't know

We operationalize this survey item in two different ways. First we create a binary dummy variable which is coded 1 for all those who selected anxious and zero for those who did not. Next we constructed a positive emotions binary variable which is coded one for all those respondents who selected at least one positive emotion – i.e., motivated, optimistic, hopeful – and zero for those who did not.

D.3 Measurement of Moderators

In Figure 3 in the main text we report the results a series of models testing for heterogenous treatment effects across a range of moderators. We briefly here lay out how we operationalized these moderators.

Political Affiliation

We define a binary variable labelled "conservative" based on whether a respondent feels closest to either a center-right or right wing party (1) or other parties to the left (0).

Personal Cost

Personal cost is coded differently depending on which outcome is being tested. For the beef tax and meat-free canteen outcomes we use a variable which asks respondents who much red meat they consume weekly. We define those above the mean as facing a high cost (1) and the rest as facing low cost (0). For the flight ban we use a variable on early flights consumption within Europe in the previous year. This variable is not normally distributed and as such we define those in the upper quartile as facing high cost (1) and those below as facing low cost (0).

Trust in Government

We use a proxy measure capturing agreement about whether there is corruption in French national public institutions to construct a binary variable where respondents are coded as high trust (1)

when they perceive low levels of corruption (1–3 on a Likert scale) and low trust when they perceive high levels of corruption (4-5 on a Likert scale)

Activism Index

We asked respondents whether they had ever engaged in a number of different forms of activism, i.e., participating in climate demonstrations, donating money to an environmental organization, or shared content with others to raise climate awareness. If a respondent reports engaging in at least one of these activities we code them as high on our activism index (1) and low (0) otherwise.

Gender

Gender is coded as a binary variable where Female = 1 and Male = 0.

Income

We define income based on the income of a respondents highest-earning parent. We take the median value and define those above as being high income (1) and those below as being low income (0).

Behavioral Measures

At the end of our survey, participants were offered the option to enter a lottery with a €100 prize (conditional on also completing a follow-up survey six weeks after the workshop). If they opted in to the lottery, they had the option to donate a portion of their potential prize winnings to *Réseau Action Climat*, an environmental NGO which does advocacy work on the topics of the policies we ask about earlier in the survey. Participants were informed that, if they win the lottery, they would retain any portion of the prize money not donated on the condition that they respond to the endline survey, which is distributed to them via email six weeks after the completion of the workshop. Donation allocations are made using a sliding scale, with €1 increments ranging from €0 to €100, and the default set at €0. Participants were told that the winner will be contacted at the conclusion of our study in Spring 2025 and are given no information about how many participants we expect to reach.

The donation approach is inspired by incentivised dictator games which have been used in economics and political science literatures to measure outcomes such as risk preferences [7], altruism [8, 9] and co-ethnic biases in social interactions [10, 11]. Our €100 prize is substantially higher than amounts offered in some studies in the literature [12]. However in the context of our lottery-based design, participants may estimate their chances of winning the lottery to be low. This could bias our results in an upward direction, since participants are more willing to give away money which they think they are unlikely to win. However, this could also downward bias the results, if participants see little benefit to making hypothetical donations.

E Further Results

E.1 Persistence of effects

Research Design

To test the persistence effects six weeks after the workshop we use two empirical strategies. First, using a within-group design, we estimate the following:

$$Y_{it} = \beta_0 + \beta_1 T + \theta_i + \delta_w + \epsilon_{it} \tag{1}$$

where i is the respondent, T is a dummy indicating whether an individual's response was recorded at time of the workshop or 6 weeks later, and Y is the outcome of interest. We use an individual fixed effect (θ), a workshop fixed effect (α_w) and cluster-robust standard errors CR2 at the individual level. β_1 is the average treatment effect we are interested in.

Next, using a between-group design, we estimate a difference-in-differences specification that interacts treatment status and survey wave (workshop day vs. endline). This approach compares changes in support for costly climate policies between treated and control respondents over time. The specification is:

$$Y_{it} = \beta_0 + \beta_1 Z_i + \beta_2 Post_t + \beta_3 (Z_i \times Post_t) + \theta_u + \epsilon_{it} \tag{2}$$

where Z_i is a treatment indicator, $Post_t$ is a post-treatment dummy (equal to 1 for endline responses), and θ_u are university fixed effects. The coefficient of interest is β_3, which captures the persistence of treatment effects relative to the control group. Standard errors are clustered at the workshop level.

Results – Within-Group

Table 8 reports the results of the within-group analysis estimating medium-term changes in policy support among respondents who were in the control group at baseline (i.e., those who had not yet participated in the workshop at the time of the initial survey). These models include individual fixed effects and therefore identify changes in support within the same respondent between the initial and endline surveys. The key coefficient is the indicator for the six-week follow-up ("Time"), which captures the average within-person change in support over this period.

We find positive and statistically significant within-individual increases in support for a beef tax and a meat-free canteen, while the estimated change for support for a flight ban is small and not statistically distinguishable from zero. These results suggest an upward shift in support among

baseline control respondents over the six-week period, in line with our main results. Because these individuals were treated by the time of the endline survey, these within-person increases provide further evidence that the effect of workshop participation on policy support persists over the medium term.

However, these estimates should be interpreted with caution given the relatively small number of respondents observed at both time points and the limited statistical power of the within-person design. Nonetheless, the pattern of results is directionally consistent with the following between-group analysis and supports the conclusion that the effects of the workshop are not short-lived.

Table 8. Persistence of the effects of workshop participation on support for costly climate policies (Within-Analysis)

	**Policy support (in %)**		
	Beef tax	Flight ban	Meat-free canteen
Time (6 weeks)	0.22***	0.04	0.13**
	(0.03)	(0.06)	(0.04)
Adj. R^2	0.18	0.01	0.07
Num. obs.	306	308	290
N Clusters	123	123	123

Note: Coefficients are from within-individual fixed-effects models using only respondents in the control arm at baseline. The coefficient on *Time* reflects the average within-person change in support over the six-week period. Standard errors are clustered at the individual level. Outcome variables are binary indicators coded 1 if respondents rated support for a given policy as 4 or 5 on a 5-point Likert scale. $^{***}p < 0.001$; $^{**}p < 0.01$; $^{*}p < 0.05$; $^{\dagger}p < 0.1$.

Results – Between-Group

Table 9 shows the results of the between-group analysis examining the long-term effect of workshop participation on our three policy support outcomes. The models include an interaction between a treatment indicator (treatment or control during the workshop) and a time indicator (referring to the initial survey or the endline survey). The coefficient on the treatment variable reflects the difference in support in the initial survey between the treatment and control groups. The post-survey coefficient captures the change in support over time within the control group. The interaction term shows how the change in support over time in the treatment group differs from the change in the control group, i.e. the difference-in-differences estimate.

The positive interaction coefficients for the beef tax and meat-free canteen policies suggest that the

increase in support among control participants was larger compared with treatment participants. This pattern is driven primarily by an increase in support for costly climate policies in the control group rather than a decline in the treatment group. This in turn indicates that the treatment persists over the medium-term, as, due to the nature of our design, the control group have been treated by the time we administered the endline survey six weeks later. The estimated treatment effects are large, but should be interpreted with caution given the small sample size and relatively large standard errors, which introduce uncertainty.

Table 9. Persistence of the effects of workshop participation on support for costly climate policies (Between-Analysis)

	**Policy support (in %)**		
	Beef tax	Flight ban	Meat-free canteen
Treatment	-0.26^{***}	-0.07	-0.10
	(0.06)	(0.05)	(0.06)
Post-Survey (6 weeks)	0.02	0.02	-0.03
	(0.04)	(0.04)	(0.05)
Treatment × Post-Survey	0.20^{***}	0.03	0.19^{**}
	(0.05)	(0.05)	(0.06)
Adj. R^2	0.12	0.05	0.08
Num. obs.	635	638	620
N Clusters	123	123	123

Note: Results from difference-in-differences models interacting a treatment indicator (treated at the workshop) with a time indicator (initial survey vs. endline). Models include experimental block fixed effects and are clustered at the workshop level (123 clusters). Outcome variables are binary indicators equal to 1 if respondents rated support as 4 or 5 on the 5-point scale. $^{***}p < 0.001$; $^{**}p < 0.01$; $p < 0.05$; $^{\dagger}p < 0.1$.

E.2 Main Results – Support for Costly Climate Policies

Beef Tax

Table 10. Effects of workshop participation on support for a beef tax (doubling the price of beef)

	**Immediate Effect**	**Persistence Effects**	
		6-Week (Between)	6-Week (Within)
Treatment	0.07^{**}	-0.26^{***}	–
	(0.02)	(0.06)	
Post-Survey (6 wks)	–	0.02	–
		(0.04)	
Treatment × Post-Survey	–	0.20^{***}	–
		(0.05)	
Time (6 wks)	–	–	0.22^{***}
			(0.03)
Adj. R^2	0.06	0.12	0.18
Num. obs.	1800	635	306
Clusters	167	123	123
Control mean	0.34	–	–
Block FE	Yes	Yes	Yes
Individual FE	No	No	Yes
Estimator	ITT	DiD	Within-FE

Note: Immediate effects are ITT estimates with block fixed effects and standard errors clustered at the workshop level. The 6-week (between) column reports coefficients from a difference-in-differences model with block fixed effects and cluster-robust standard errors at the workshop level (123 clusters). The 6-week (within) column reports within-individual fixed-effects estimates among baseline control respondents, with standard errors clustered at the individual level. Outcome variables are binary indicators coded 1 if respondents rated support for a given policy as 4 or 5. $^{***}p < 0.001$; $^{**}p < 0.01$; $^{*}p < 0.05$; $^{\dagger}p < 0.1$.

Flight Ban

Table 11. Effects of workshop participation on support for a flight ban (on journeys for which a six-hour or less train journey is available)

	**Immediate Effect**	**Persistence Effects**	
		6-Week (Between)	6-Week (Within)
Treatment	0.07**	−0.07	–
	(0.02)	(0.05)	
Post-Survey (6 wks)	–	0.02	–
		(0.04)	
Treatment × Post-Survey	–	0.03	–
		(0.05)	
Time (6 wks)	–	–	0.04
			(0.06)
Adj. R^2	0.14	0.05	0.01
Num. obs.	1810	638	308
Clusters	167	123	123
Control mean	0.55	–	–
Block FE	Yes	Yes	Yes
Individual FE	No	No	Yes
Estimator	ITT	DiD	Within-FE

Note: Immediate effects are ITT estimates with block fixed effects and standard errors clustered at the workshop level. The 6-week (between) column reports coefficients from a difference-in-differences model with block fixed effects and cluster-robust standard errors at the workshop level (123 clusters). The 6-week (within) column reports within-individual fixed-effects estimates among baseline control respondents, with standard errors clustered at the individual level. Outcome variables are binary indicators coded 1 if respondents rated support for a given policy as 4 or 5. $^{***}p < 0.001$; $^{**}p < 0.01$; $^{*}p < 0.05$; $^{\dagger}p < 0.1$.

Meat-Free Canteen

Table 12. Effects of workshop participation on support for a meat-free canteen

	**Immediate Effect**	**Persistence Effects**	
		6-Week (Between)	6-Week (Within)
Treatment	0.07**	−0.10	–
	(0.02)	(0.06)	
Post-Survey (6 wks)	–	−0.03	–
		(0.05)	
Treatment × Post-Survey	–	0.19**	–
		(0.06)	
Time (6 wks)	–	–	0.13**
			(0.04)
Adj. R^2	0.05	0.08	0.07
Num. obs.	1818	620	290
Clusters	167	123	123
Control mean	0.36	–	–
Block FE	Yes	Yes	Yes
Individual FE	No	No	Yes
Estimator	ITT	DiD	Within-FE

Note: Immediate effects are ITT estimates with block fixed effects and standard errors clustered at the workshop level. The 6-week (Between) column reports coefficients from a difference-in-differences model with block fixed effects and cluster-robust standard errors at the workshop level (123 clusters). The 6-week (Within) column reports within-individual fixed-effects estimates among baseline control respondents, with standard errors clustered at the individual level. Outcome variables are binary indicators coded 1 if respondents rated support for a given policy as 4 or 5. $^{***}p < 0.001$; $^{**}p < 0.01$; $^{*}p < 0.05$; $^{\dagger}p < 0.1$.

Power Calculations – Main Effects

We conducted ex-post power calculations using the EGAP Power Calculator for cluster-randomized experiments. We base our calculations on actual figures of 83 clusters per treatment arm, a two-sided significance level of 0.05, and calculated intra-cluster correlation coefficients (ICCs) ranging from 0.02 to 0.03. The outcomes are binary indicators of support for the three climate policy outcomes we study.

Table 13. Power calculations for primary outcomes

Outcome	Control	Treatment	ICC	Clusters/arm	$N_{0.8}$	Power ($N = 1845$)
Beef tax	0.343	0.418	0.02	83	1544	0.855
Meat-free canteen	0.358	0.434	0.03	83	1662	0.830
Flight ban	0.552	0.625	0.02	83	1702	0.825

Notes: Control and treatment means reflect the proportions of respondents expressing support for each policy. Effect sizes are absolute differences in proportions (percentage points). Power calculations assume a two-sided test with $\alpha = 0.05$, cluster-level random assignment, and equal cluster sizes. $N_{0.8}$ denotes the total sample size required to achieve 80% power.

The calculations reflect actual control and treatment-group support levels. Under these conditions, the total sample size required to achieve 80 percent statistical power ranges from 1,544 to 1,702 respondents across outcomes. Given our total sample size of 1,845 respondents, the study achieves power between 0.83 and 0.86 for all three outcomes. The flight ban outcome represents the most demanding case in terms of statistical power, but the planned sample size exceeds the minimum required to achieve 80 percent power for all outcomes. Overall, the study is adequately powered.

E.3 Mechanisms / Manipulation Checks

Mediation Analysis

We conduct causal mediation analyses within the counterfactual (potential outcomes) framework of Imai, Keele, and Tingley (2010) [13]. Specifically, we estimate the Average Causal Mediation Effect (ACME), which represents the average indirect effect of the treatment on the outcome operating through the mediator. We also estimate the Average Direct Effect (ADE), defined as the average effect of the treatment on the outcome that is not transmitted through the mediator. Confidence intervals are obtained via non-parametric bootstrap with clustering at the workshop level. Identification relies on the sequential ignorability assumption. While random assignment satisfies the ignorability of treatment, identification further requires that, conditional on treatment and observed covariates, there are no unobserved factors that jointly influence the mediator and the outcome.

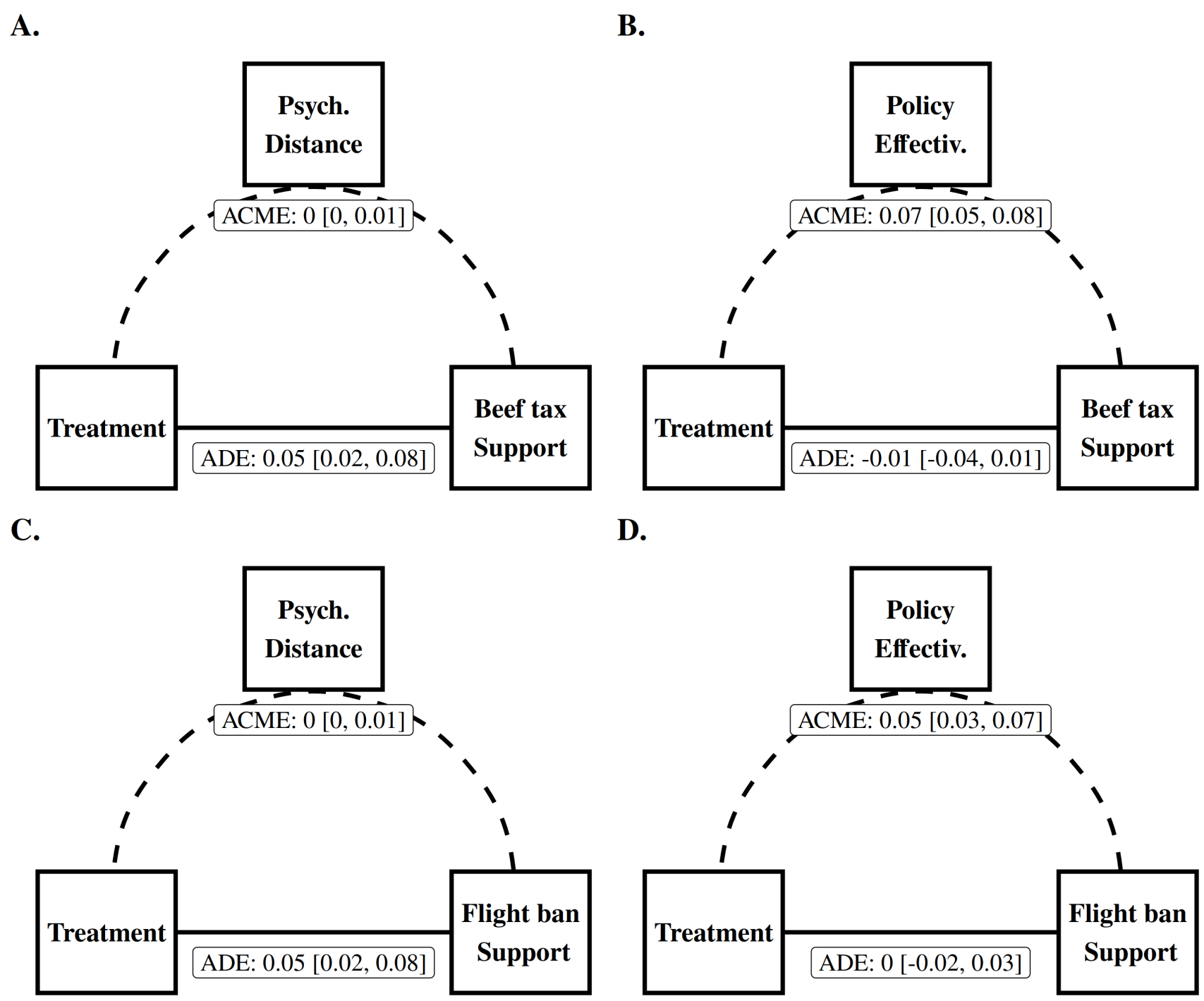

Figure 2. Causal mediation analysis of workshop effects

Notes: Panels A–D display path diagrams from causal mediation models estimating whether the treatment effect on policy support operates through (i) perceived psychological distance or (ii) perceived policy effectiveness, for beef tax support (A–B) and flight ban support (C–D). Mediation analyses are implemented using the `mediate` function with 1,000 quasi-Bayesian Monte Carlo simulations. The solid arrow denotes the average direct effect (ADE) of treatment on policy support; the dashed curved arrow denotes the average causal mediation effect (ACME). Boxes report point estimates with 95% confidence intervals. Mediator models regress the mediator on treatment and university fixed effects; outcome models regress standardized policy support on treatment, the mediator, and university fixed effects. Estimates are based on complete-case observations for each model.

Sensitivity Analysis

To assess the robustness of our mediation results, we conduct a formal sensitivity analysis following Imai, Keele, and Tingley (2010) [13]. The sensitivity analysis evaluates how strong unobserved mediator–outcome confounding would need to be to eliminate the estimated indirect effects.

Table 14. Sensitivity Analysis for the Average Causal Mediation Effect (ACME)

Mediator → Outcome	ACME	95% CI	p	ρ^*	$R^2_M R^2_Y$
Psych. distance → Beef tax support	0.002	[-0.005, 0.009]	0.518	0.20	0.04
Policy effectiveness → Beef tax support	0.067	[0.051, 0.084]	< 0.001	0.50	0.25
Psych. distance → Flight-ban support	0.001	[-0.004, 0.007]	0.632	0.20	0.04
Policy effectiveness → Flight-ban support	0.052	[0.033, 0.072]	< 0.001	0.60	0.36

Notes: ρ^* denotes the value of the sensitivity parameter (the correlation between mediator and outcome residuals) at which the ACME equals zero. $R^2_M R^2_Y$ denotes the product of the proportions of residual variance in the mediator and outcome models that an omitted confounder would need to explain to eliminate the indirect effect.

The parameter ρ represents the correlation between the unobserved components of the mediator and outcome models. Under the identifying assumption of sequential ignorability, $\rho = 0$. Larger values of ρ indicate stronger unobserved confounding.

For mediation via psychological distance, the ACME is small and statistically indistinguishable from zero in both policy domains. The indirect effect reaches zero at $\rho \approx 0.20$, corresponding to a product of residual variance explained of approximately 0.04. This indicates that even relatively weak unobserved confounding would suffice to eliminate the already small mediated effect.

By contrast, mediation via perceived policy effectiveness is substantively larger and highly statistically significant for both beef tax support and flight-ban support. The ACME would be reduced to zero only at $\rho \approx 0.50$ (beef) and $\rho \approx 0.60$ (flight), corresponding to products of residual variance explained of 0.25 and 0.36, respectively. These values imply that an omitted confounder would need to explain a substantial share of residual variation in both the mediator and outcome models to overturn the estimated indirect effects.

Taken together, the sensitivity analysis suggests that the mediation effects operating through perceived policy effectiveness are comparatively robust to unobserved confounding, whereas mediation via psychological distance is weak and not robust.

Effectiveness Beliefs – Beef Tax

Table 15. Effects of workshop participation on perceived effectiveness of a beef tax

	**Immediate Effect**	**Persistence Effects**	
		6-Week (Between)	6-Week (Within)
Treatment	0.18^{***}	-0.23^{***}	–
	(0.02)	(0.05)	
Post-Survey (6 wks)	–	-0.09^{*}	–
		(0.04)	
Treatment × Post-Survey	–	0.15^{*}	–
		(0.06)	
Time (6 wks)	–	–	0.07^{*}
			(0.03)
Adj. R^2	0.05	0.09	0.02
Num. obs.	1804	637	308
Clusters	167	123	123
Control mean	0.51	–	–
Block FE	Yes	Yes	Yes
Individual FE	No	No	Yes
Estimator	ITT	DiD	Within-FE

Note: Immediate effects are ITT estimates with block fixed effects and standard errors clustered at the workshop level. The 6-week (Between) column reports coefficients from a difference-in-differences model with block fixed effects and cluster-robust standard errors at the workshop level (123 clusters). The 6-week (Within) column reports within-individual fixed-effects estimates among baseline control respondents, with standard errors clustered at the individual level. Outcome variables are binary indicators coded 1 if respondents rated the policy as effective (4 or 5). $^{***}p < 0.001$; $^{**}p < 0.01$; $^{*}p < 0.05$; $^{\dagger}p < 0.1$.

Effectiveness Beliefs – Flight Ban

Table 16. Effects of workshop participation on perceived effectiveness of a flight ban

	**Immediate Effect**	**Persistence Effects**	
		6-Week (Between)	6-Week (Within)
Treatment	0.10***	−0.09†	–
	(0.02)	(0.05)	
Post-Survey (6 wks)	–	−0.03	–
		(0.03)	
Treatment × Post-Survey	–	0.03	–
		(0.04)	
Time (6 wks)	–	–	0.01
			(0.04)
Adj. R^2	0.09	0.04	0.01
Num. obs.	1809	635	304
Clusters	167	123	123
Control mean	0.67	–	–
Block FE	Yes	Yes	Yes
Individual FE	No	No	Yes
Estimator	ITT	DiD	Within-FE

Note: Immediate effects are ITT estimates with block fixed effects and standard errors clustered at the workshop level. The 6-week (Between) column reports coefficients from a difference-in-differences model with block fixed effects and cluster-robust standard errors at the workshop level (123 clusters). The 6-week (Within) column reports within-individual fixed-effects estimates among baseline control respondents, with standard errors clustered at the individual level. Outcome variables are binary indicators coded 1 if respondents rated the policy as effective (4 or 5). $^{***}p < 0.001$; $^{**}p < 0.01$; $^{*}p < 0.05$; $^{\dagger}p < 0.1$.

Psychological Distance

Table 17. Effects of workshop participation on psychological distance

	**Immediate Effect**	**Persistence Effects**	
		6-Week (Between)	6-Week (Within)
Treatment	0.01	−0.03	–
	(0.01)	(0.02)	
Post-Survey (6 wks)	–	−0.00	–
		(0.02)	
Treatment × Post-Survey	–	0.01	–
		(0.02)	
Time (6 wks)	–	–	0.01
			(0.01)
Adj. R^2	0.04	0.13	0.01
Num. obs.	1835	641	314
Clusters	167	123	123
Control mean	0.70	–	–
Block FE	Yes	Yes	Yes
Individual FE	No	No	Yes
Estimator	ITT	DiD	Within-FE

Note: Immediate effects are ITT estimates with block fixed effects and standard errors clustered at the workshop level. The 6-week (Between) column reports coefficients from a difference-in-differences model with block fixed effects and cluster-robust standard errors at the workshop level (123 clusters). The 6-week (Within) column reports within-individual fixed-effects estimates among baseline control respondents, with standard errors clustered at the individual level. Psychological distance is a single Likert item rescaled to the range 0–1. $^{***}p < 0.001$; $^{**}p < 0.01$; $^{*}p < 0.05$; $^{\dagger}p < 0.1$.

Knowledge

Table 18. Effects of workshop participation on climate knowledge

	**Immediate Effect**	**Persistence Effects**	
		6-Week (Between)	6-Week (Within)
Treatment	0.22***	−0.44***	–
	(0.02)	(0.05)	
Post-Survey (6 wks)	–	−0.19***	–
		(0.04)	
Treatment × Post-Survey	–	0.33***	–
		(0.07)	
Time (6 wks)	–	–	0.14*
			(0.06)
Adj. R^2	0.11	0.12	0.05
Num. obs.	1832	637	306
Clusters	167	123	123
Control mean	0.49	–	–
Block FE	Yes	Yes	Yes
Individual FE	No	No	Yes
Estimator	ITT	DiD	Within-FE

Note: Immediate effects are ITT estimates with block fixed effects and workshop-clustered standard errors. The 6-week (Between) estimates are from difference-in-differences models with block fixed effects and workshop-clustered standard errors (123 clusters). The 6-week (Within) estimates come from within-individual fixed-effects models among baseline control respondents, with standard errors clustered at the individual level. For the immediate effect, climate knowledge is the share of correct responses to two carbon-footprint questions. For the endline survey, we only asked one of these two questions, meaning this measure is based on the answer to only one question (see SI Section D.2). $^{***}p < 0.001$; $^{**}p < 0.01$; $^{*}p < 0.05$; $^{\dagger}p < 0.1$.

Emotions − Anxiety

Table 19. Effects of workshop participation on anxiety

	**Immediate Effect**	**Persistence Effects**	
		6-Week (Between)	6-Week (Within)
Treatment	−0.00	−0.01	–
	(0.02)	(0.05)	
Post-Survey (6 wks)	–	0.07	–
		(0.04)	
Treatment × *Post-Survey*	–	0.00	–
		(0.06)	
Time (6 wks)	–	–	0.06
			(0.04)
Adj. R^2	0.03	0.08	0.02
Num. obs.	1844	642	314
Clusters	167	123	123
Control mean	0.21	–	–
Block FE	Yes	Yes	Yes
Individual FE	No	No	Yes
Estimator	ITT	DiD	Within-FE

Note: Immediate effects are ITT estimates with block fixed effects and clustered SEs at the workshop level. The 6-week (Between) estimates are from DiD models with block fixed effects and workshop-clustered SEs (123 clusters). The 6-week (Within) estimates come from within-individual fixed-effects models among baseline control respondents, with SEs clustered at the individual level. Anxiety = binary variable indicating whether respondents picked anxiety from a choice list when asked about their reaction to climate change. $^{***}p < 0.001$; $^{**}p < 0.01$; $^{*}p < 0.05$; $^{\dagger}p < 0.1$.

Emotions – Positive

Table 20. Effects of workshop participation on positive emotions

	Immediate Effect	Persistence Effects	
		6-Week (Between)	6-Week (Within)
Treatment	0.08***	−0.09†	–
	(0.02)	(0.05)	
Post-Survey (6 wks)	–	−0.16***	–
		(0.04)	
Treatment × Post-Survey	–	0.15**	–
		(0.06)	
Time (6 wks)	–	–	−0.01
			(0.03)
Adj. R^2	0.03	0.09	0.01
Num. obs.	1845	658	330
Clusters	167	123	123
Control mean	0.37	–	–
Block FE	Yes	Yes	Yes
Individual FE	No	No	Yes
Estimator	ITT	DiD	Within-FE

Note: Immediate effects are ITT estimates with block fixed effects and clustered SEs at the workshop level. The 6-week (Between) estimates are from DiD models with block fixed effects and workshop-clustered SEs (123 clusters). The 6-week (Within) estimates come from within-individual fixed-effects models among baseline control respondents, with SEs clustered at the individual level. Positive emotions is a binary variable coded 1 if respondents selected at least one positive emotion (motivated, optimistic, hopeful) from the list of options and 0 otherwise. $^{***}p < 0.001$; $^{**}p < 0.01$; $^{*}p < 0.05$; $^{\dagger}p < 0.1$.

E.4 Moderators

Conservative Ideology

Table 21. Heterogeneous treatment effects by political affiliation (Conservative)

	**Beef tax**	**Flight ban**	**Meat-free canteen**
Treatment	0.08**	0.07**	0.09***
	(0.02)	(0.02)	(0.03)
Conservative	-0.15***	-0.11*	-0.13**
	(0.04)	(0.05)	(0.04)
Treatment × *Conservative*	-0.08	0.02	-0.11*
	(0.05)	(0.06)	(0.05)
Adj. R^2	0.08	0.14	0.07
Num. obs.	1800	1810	1818
Clusters	167	167	167
Block FE	Yes	Yes	Yes
Estimator	ITT	ITT	ITT

Note: Estimates from ITT models with fixed effects for universities and experimental blocks. Standard errors are clustered at the workshop level (167 clusters). "Treatment × Conservative" reports the differential treatment effect for respondents who identify as conservative. Conservative: coded 1 if the respondent identifies with a center-right or right-wing party, 0 otherwise. $^{***}p < 0.001$; $^{**}p < 0.01$; $^{*}p < 0.05$; $^{\dagger}p < 0.1$.

Personal Cost

Table 22. Heterogeneous treatment effects by personal cost

	**Beef tax**	**Flight ban**	**Meat-free canteen**
Treatment	0.08^{**}	0.05^{*}	0.08^{*}
	(0.03)	(0.02)	(0.03)
High personal cost	-0.20^{***}	-0.18^{***}	-0.17^{***}
	(0.03)	(0.03)	(0.03)
Treatment × *High cost*	-0.02	0.05	-0.01
	(0.04)	(0.05)	(0.05)
Adj. R^2	0.10	0.16	0.08
Num. obs.	1800	1810	1818
Clusters	167	167	167
Block FE	Yes	Yes	Yes
Estimator	ITT	ITT	ITT

Note: Estimates from ITT models with university and experimental block fixed effects. Standard errors are clustered at the workshop level (167 clusters). "Treatment × High cost" reports the interaction between treatment and a dummy for respondents in the top quartile (flight consumption) or above the mean (beef consumption) of personal costs. $^{***}p < 0.001$; $^{**}p < 0.01$; $^{*}p < 0.05$; $^{\dagger}p < 0.1$.

Trust in Government

Table 23. Heterogeneous treatment effects by trust in government

	**Beef tax**	**Flight ban**	**Meat-free canteen**
Treatment	0.02	0.08	$0.11^{\dagger}$
	(0.06)	(0.05)	(0.06)
Low Trust	0.03	0.02	0.05
	(0.05)	(0.05)	(0.04)
Treatment × *Low Trust*	0.07	-0.01	-0.07
	(0.07)	(0.07)	(0.06)
Adj. R^2	0.06	0.11	0.05
Num. obs.	1232	1230	1233
Clusters	166	166	166
Block FE	Yes	Yes	Yes
Estimator	ITT	ITT	ITT

Note: Estimates from ITT models with university and experimental block fixed effects. Standard errors are clustered at the workshop level (166 clusters). "Treatment × Low Trust" reports the conditional effect of treatment among respondents who express low trust in government via our proxy measure (responses 4–5 on the 5-point corruption scale). $^{***}p < 0.001$; $^{**}p < 0.01$; $^{*}p < 0.05$; $^{\dagger}p < 0.1$.

Prior Activism

Table 24. Heterogeneous treatment effects by activism level

	**Beef tax**	**Flight ban**	**Meat-free canteen**
Treatment	0.06*	0.04	0.09**
	(0.03)	(0.02)	(0.03)
High Activism	0.00	0.01	0.05
	(0.03)	(0.03)	(0.04)
Treatment × High Activism	0.03	0.09*	-0.04
	(0.05)	(0.04)	(0.05)
Adj. R^2	0.06	0.14	0.05
Num. obs.	1800	1810	1818
Clusters	167	167	167
Block FE	Yes	Yes	Yes
Estimator	ITT	ITT	ITT

Note: ITT estimates with fixed effects for universities and experimental blocks. Standard errors are clustered at the workshop level (167 clusters). "Treatment × High Activism" reports the conditional effect of treatment among respondents scoring 1 on the activism index. $^{***}p < 0.001$; $^{**}p < 0.01$; $^{*}p < 0.05$; $^{\dagger}p < 0.1$.

Gender

Table 25. Heterogeneous treatment effects by gender

	**Beef tax**	**Flight ban**	**Meat-free canteen**
Treatment	$0.06^{\dagger}$	0.06^{*}	0.11^{***}
	(0.03)	(0.03)	(0.03)
Female	0.14^{***}	-0.01	0.19^{***}
	(0.04)	(0.03)	(0.03)
Treatment × *Female*	0.02	0.01	-0.07
	(0.05)	(0.04)	(0.04)
Adj. R^2	0.08	0.13	0.07
Num. obs.	1784	1794	1802
Clusters	167	167	167
Block FE	Yes	Yes	Yes
Estimator	ITT	ITT	ITT

Note: ITT estimates with fixed effects for universities and experimental blocks. Standard errors clustered at the workshop level (167 clusters). "Treatment × Female" reports the conditional effect of treatment among women (female = 1). $^{***}p < 0.001$; $^{**}p < 0.01$; $^{*}p < 0.05$; $^{\dagger}p < 0.1$.

Income

Table 26. Heterogeneous treatment effects by income

	**Beef tax**	**Flight ban**	**Meat-free canteen**
Treatment	0.10^{*}	0.09^{**}	0.05
	(0.04)	(0.03)	(0.04)
High Income	0.07^{*}	-0.02	$-0.07^{\dagger}$
	(0.03)	(0.04)	(0.04)
Treatment × *High Income*	-0.09	-0.05	0.05
	(0.05)	(0.05)	(0.05)
Adj. R^2	0.06	0.13	0.05
Num. obs.	1410	1415	1418
Clusters	167	167	167
Block FE	Yes	Yes	Yes
Estimator	ITT	ITT	ITT

Note: ITT estimates with university and block fixed effects. Standard errors clustered at the workshop level (167 clusters). "Treatment × High Income" gives the conditional treatment effect among respondents whose parents earn above the median income. $^{***}p < 0.001$; $^{**}p < 0.01$; $^{*}p < 0.05$; $^{\dagger}p < 0.1$.

Power Calculations – Heterogenous Effects

Table 27. Minimum detectable heterogeneous treatment effects

Outcome	Control mean	ICC	Clusters/arm	N	Main MDE (pp)	Heterogeneity MDE (pp)
Beef tax	0.343	0.022	83	1800	6.92	9.79
Flight ban	0.552	0.015	83	1810	7.03	9.94
Meat-free canteen	0.358	0.026	83	1818	7.06	9.99

Notes: Entries report minimum detectable effects (MDEs) in percentage points for a two-sided test with $\alpha = 0.05$ and statistical power of 0.80. Outcomes are binary indicators of policy support. Intracluster correlations (ICCs) are estimated from mixed-effects models conditioning on university and experimental block fixed effects. Effective sample sizes incorporate standard design-effect adjustments for clustered assignment. Main MDE denotes the detectable average treatment effect. Heterogeneity MDE denotes the minimum detectable difference in treatment effects between two equally sized subgroups, computed using a conservative $\sqrt{2}$ variance inflation factor.

To assess statistical power to detect heterogeneous treatments effects we calculated minimum detectable effects (MDEs) using a standard formula for blocked cluster-randomized experiments. All calculations were based on binary outcomes measures of policy support, coded as 1 if a respondent supports a policy, and 0 otherwise. Outcome variance is computed from control-group means. Intra-cluster correlations are estimated from mixed-effects models conditioning on university and experimental block fixed effects, and effective sample sizes are obtained using standard design-effect adjustments.

Power for heterogeneous treatment effects is assessed using a conservative approximation [14]. Because heterogeneity corresponds to differences between subgroup-specific treatment effects (i.e., interaction terms), the associated MDEs are inflated by a factor of $\sqrt{2}$ relative to the average treatment effect, reflecting the greater sampling variance of interaction estimators. This approach provides a lower bound on power for heterogeneity analyses.

Table 27 reports the estimates. Under the study design, the MDE of the main specification at 80% percent power is approximately 7 percentage points across all outcomes, in line with our actual observed estimates. The corresponding MDE for heterogeneous effects – defined as the difference in treatment effects between two equally sized subgroups – is approximately 9–10 percentage points.

These thresholds provide a useful benchmark for interpreting the heterogeneity analyses presented in the main text. Subgroup differences that reach statistical significance are generally of this magnitude, whereas smaller subgroup contrasts fall below the detectable range under conservative assumptions. The absence of statistically significant heterogeneity for several outcomes is therefore consistent with the limited statistical power to detect subgroup differences of smaller size, rather than definitive evidence of homogeneous treatment effects.

E.5 Behavioral Results

Lottery donations

Table 28. Causal effects of participation in the 2tonnes workshop on participation in the lottery and donation for an NGO advocating for the implementation of costly climate policy

	Lottery Participation	Donation (in €)
Treatment	0.02	−2.55
	(0.02)	(1.52)
Control mean	0.83	32
DV range	$\{0, 1\}$	$[0, 100]$
Covariates	No	No
Block FE	Yes	Yes
Estimator	ITT	ITT
Adj. R^2	0.03	0.04
Num. obs.	1845	1545
Clusters	167	167
RMSE	0.36	30.92

$^{***}p < 0.001$; $^{**}p < 0.01$; $^{*}p < 0.05$; $^{\dagger}p < 0.1$

Note: Results reported using an Intent-to-Treat (ITT) estimator, experimental block fixed effects, and cluster-robust standard errors at the workshop level. Lottery participation is measured as a binary variable. Donation is measured in euros, ranging from 0 to 100. $^{***}p < 0.001$; $^{**}p < 0.01$; $^{*}p < 0.05$; $^{\dagger}p < 0.1$.

E.6 Additional Results

Issue salience of climate change

Table 29. Effects of workshop participation on climate salience

	**Immediate Effect**	**Persistence Effects**	
		6-Week (Between)	6-Week (Within)
Treatment	0.02	$-0.11^{\dagger}$	–
	(0.02)	(0.06)	
Post-Survey (6 wks)	–	-0.01	–
		(0.05)	
Treatment × Post	–	0.08	–
		(0.07)	
Time (6 wks)	–	–	$0.08^{\dagger}$
			(0.04)
Adj. R^2	0.11	0.07	0.42
Num. obs.	1845	649	320
Clusters	167	123	123
Control mean	0.44	–	–
Block FE	Yes	Yes	Yes
Individual FE	No	No	Yes
Estimator	ITT	DiD	Within-FE

Note: Immediate effects are ITT estimates with block fixed effects and standard errors clustered at the workshop level. The 6-week (Between) specification is a DiD model with block fixed effects and clustered standard errors at the workshop level (123 clusters). The 6-week (Within) model includes individual fixed effects and SEs clustered at the individual level. $^{***}p < 0.001$; $^{**}p < 0.01$; $^{*}p < 0.05$; $^{\dagger}p < 0.1$.

Responsibility

Table 30. Causal effects of workshop participation on assessment of perceptions of whether different actors are doing enough to address climate change

	Citizens	Firms	Government
Treatment	-0.35^{***}	0.04	0.22^{*}
	(0.09)	(0.12)	(0.11)
Control mean	4.82	3.12	3.64
DV range		[0, 10]	
Block FE	Yes	Yes	Yes
Covariates	Yes	Yes	Yes
Estimator	ITT	ITT	ITT
Adj. R^2	0.02	0.10	0.09
Num. obs.	1830	1830	1830
Clusters	167	167	167
RMSE	1.90	2.33	2.24

Note: This table only reports immediate effects. Results are reported using an intent-to-treat (ITT) estimator, experimental block fixed effects, and cluster-robust standard errors at the workshop level. Outcome variables are measured on a scale from 0 to 10, which captures whether workshop participants think each actor is not doing enough (0) or doing enough (10) to address climate change. $^{***}p < 0.001$; $^{**}p < 0.01$; $^{*}p < 0.05$.

Results by university

The universities specialise in the following subjects:

- U1: social sciences, political science, public administration, and international relations
- U2: civil engineering, urban planning, environmental engineering, and transport infrastructure
- U3: social sciences, political science, public administration, and international relations
- U4: applied sciences, technology, and professional training in engineering and industry-related fields
- U5: civil aviation, air transport management, and aeronautical engineering
- U6: aerospace engineering, aeronautics, and space technology
- U7: agronomy, food science, biotechnology
- U8: veterinary sciences, agriculture, and food safety
- U9: technology, engineering, business, communication, applied sciences
- U10: business, management, finance, and marketing
- U11: civil aviation, air transport management, and aeronautical engineering

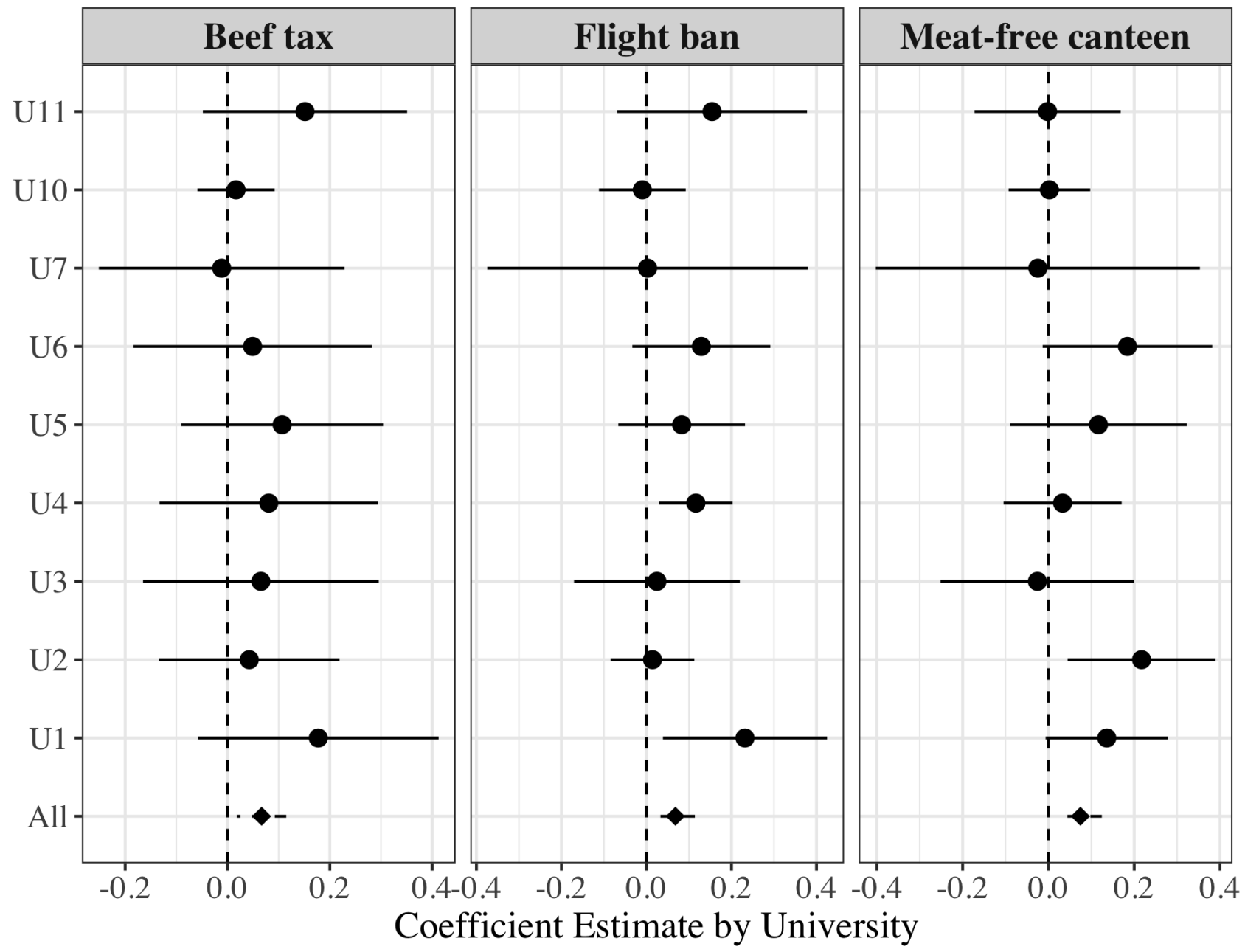

Figure 3. Estimates use a dummy measure of policy support, where responses are coded 1 if respondents show support for the policy, 0 otherwise. Dots represents the ITT estimate using block randomisation fixed effects and cluster standard errors at the workshop level. Bars represent 95% confidence interval. Two universities are excluded because of the small number of students. The final row labelled "All" shows the combined results.

Heterogeneity by discipline

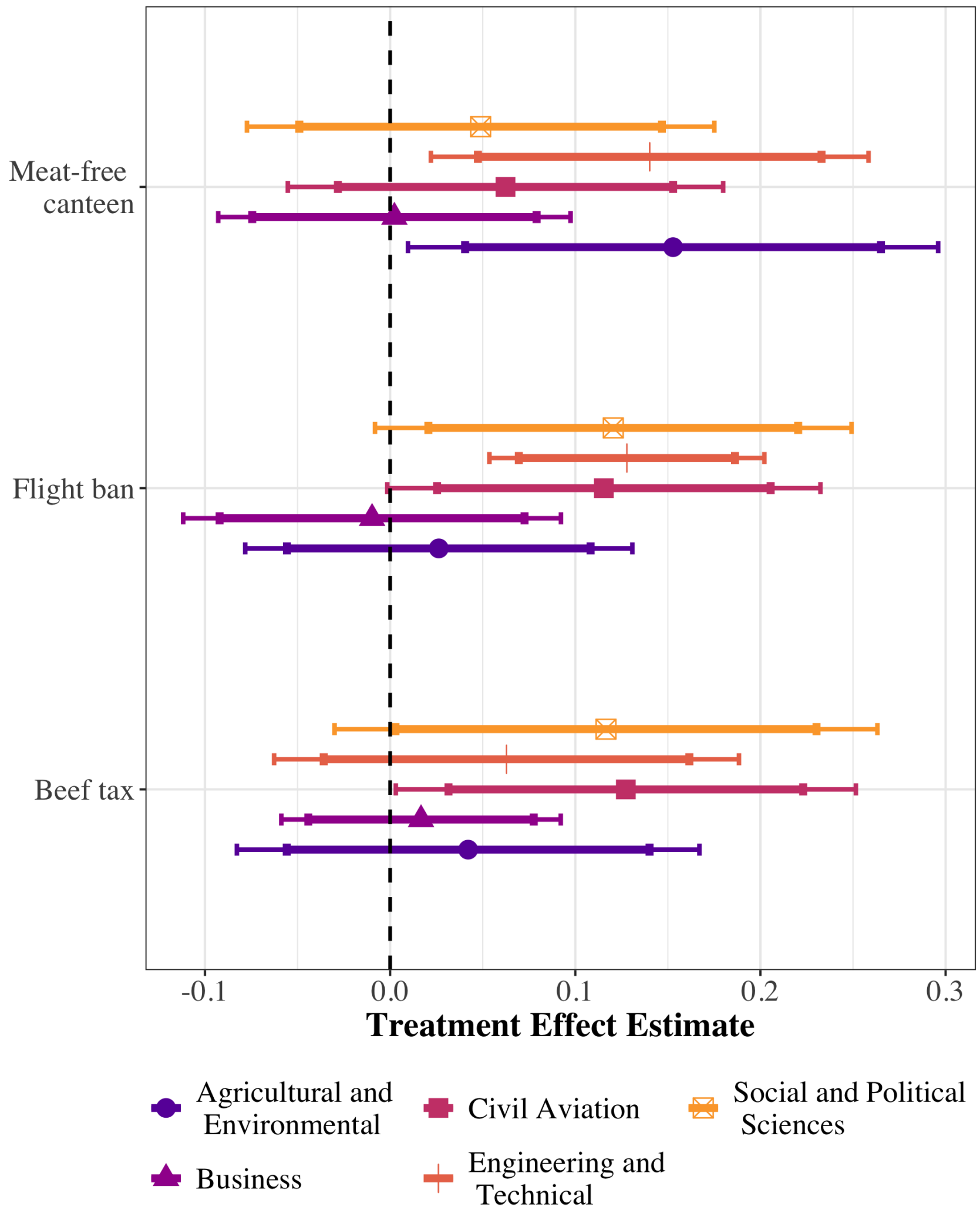

Figure 4. Estimates use a dummy measure of policy support, where responses are coded 1 if respondents show support for the policy, 0 otherwise. Dots represents the ITT estimate using block randomisation fixed effects and cluster standard errors at the workshop level. Thick bars represent 90% confidence interval and thin bars the 95% confidence interval.

F Robustness Checks

F.1 Results with Full Ordinal Scale

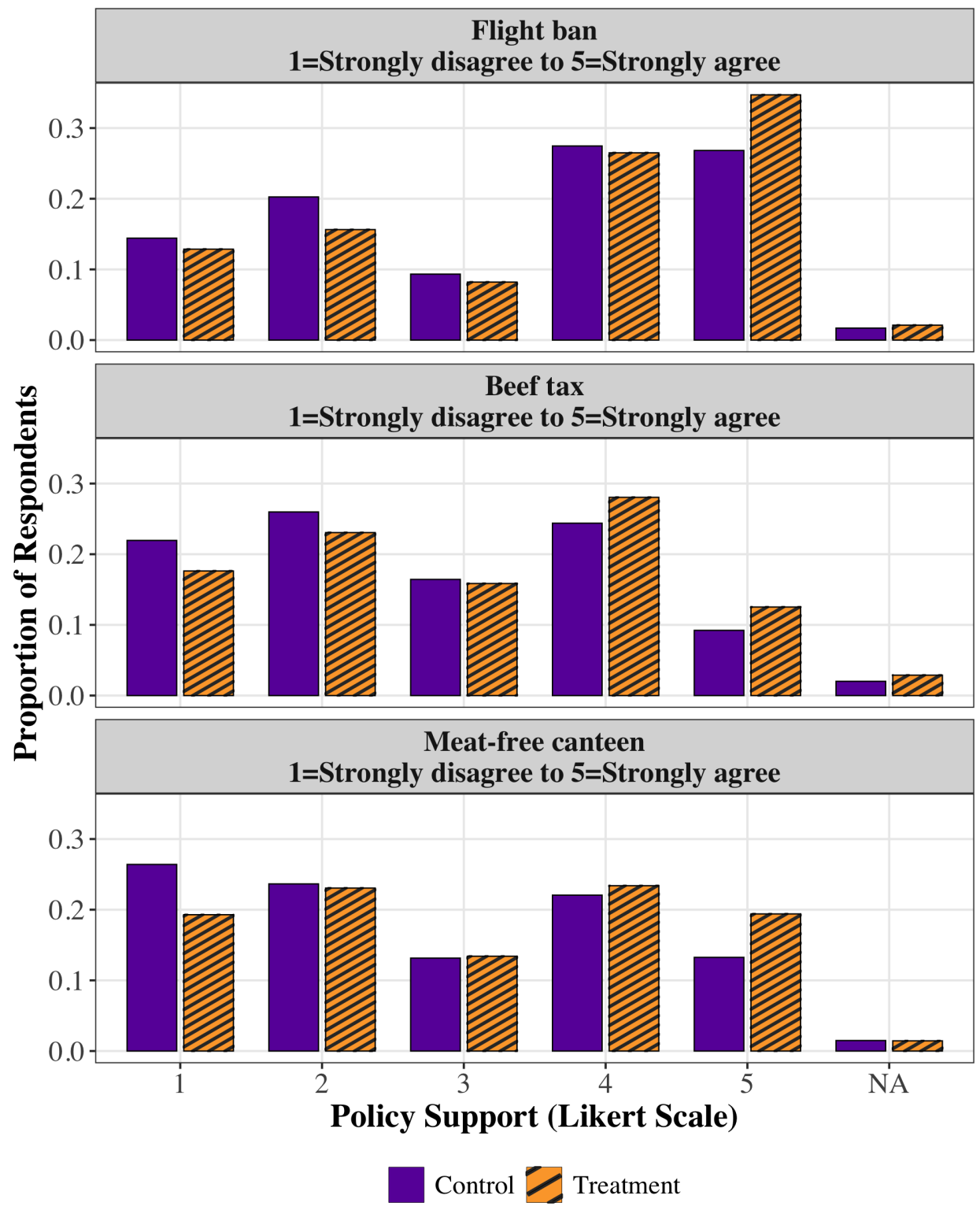

Figure 5. Distribution of policy support measured on a 5-point Likert scale (1 = strongly disagree, 5 = strongly agree).

Table 31. Causal effects of workshop participation on support for costly climate policies (5-point Likert scale)

	**Immediate effect**		
	Flight ban	Beef tax	Meat-free canteen
Treatment	0.19**	0.28***	0.21**
	(0.06)	(0.06)	(0.06)
Num. obs.	1800	1818	1810
R^2	0.10	0.11	0.20
Adj. R^2	0.07	0.08	0.17
N Clusters	167	167	167
Block FE	Yes	Yes	Yes
Estimator	ITT	ITT	ITT

Note: Coefficients are estimated using OLS regression under an intention-to-treat (ITT) framework. The outcome variables are measured on a 5-point Likert scale ranging from 1 (strongly disagree) to 5 (strongly agree), with higher values indicating greater support for each policy. All models include experimental block fixed effects. Standard errors in parentheses are clustered at the workshop level. $^{***}p < 0.001$; $^{**}p < 0.01$; $^{*}p < 0.05$; $^{\cdot}p < 0.1$.

F.2 Results from Ordered (Ordinal) Regression Models

Table 32. Causal effects of workshop participation on support for costly climate policies (ordered logit)

	**Immediate effect**		
	Flight ban	Beef tax	Meat-free canteen
Treatment	0.33***	0.29***	0.37***
	(0.08)	(0.08)	(0.08)
Num. obs.	1810	1800	1818
Log Likelihood	−2556.59	−2717.42	−2779.37
AIC	5221.18	5542.85	5666.74
BIC	5518.24	5839.60	5964.04
Block FE	Yes	Yes	Yes
Estimator	Ordered logit	Ordered logit	Ordered logit

$^{***}p < 0.001$; $^{**}p < 0.01$; $^{*}p < 0.05$; $^{\cdot}p < 0.1$

Note: Coefficients are from ordered logit models where higher values indicate greater support for the policy on a 5-point Likert scale. All specifications include experimental block fixed effects. Standard errors in parentheses are clustered at the workshop level. Across all three specifications, workshop participation is associated with significantly higher levels of support for the policy. The magnitudes of the coefficients are stable across models. Taking the exponential of each coefficient, these models indicate that treated respondents have approximately 35–45% higher odds of expressing greater support for the respective policies relative to respondents in the control group. $^{***}p < 0.001$; $^{**}p < 0.01$; $^{*}p < 0.05$; $^{\cdot}p < 0.1$.

F.3 Results with LATE estimator

Table 33. Causal effects of workshop participation on support for costly climate policies with a LATE estimator

	**Immediate effect**		
	Flight ban	Beef tax	Meat-free canteen
Treatment	0.08^{***}	0.08^{**}	0.08^{***}
	(0.02)	(0.02)	(0.02)
Num. obs.	1810	1800	1818
Adj. R^2	0.14	0.06	0.05
Block FE	Yes	Yes	Yes
Covariates	No	No	No
Estimator	LATE	LATE	LATE

Note: The immediate effect results are reported using an instrumental variable strategy where the instrument is the treatment allocation and the independent variable is the actual treatment status. It allows us to determine the LATE estimator. We use experimental block fixed effects, and cluster-robust standard errors at the workshop level. Our outcome variables are transformed from a 5-point Likert scale into a dummy variable where 1 indicates support for the policy (i.e., 4 or 5 on the Likert scale) and 0 indicates opposition or ambivalence towards the policy (i.e., 1 to 3 on the scale). $^{***}p < 0.001$; $^{**}p < 0.01$; $^{*}p < 0.05$.

F.4 Time of day

We examine whether treatment effects could be confounded by the time of day workshops are taken. In our design, this would pose an issue if there were an imbalance between the proportions of treated and control groups in the morning and afternoon sessions. As demonstrated in Table 34, most of the workshops were held in the afternoon (more than 80%). However, there is relative balance between the numbers of treated and control groups.

Table 34. Number of workshops by treatment condition and time of the day.

Afternoon	Control	Treatment
Morning	16	17
Afternoon	66	68

We examine whether the estimated treatment effects vary according to the time of day the workshops were conducted — specifically comparing morning and afternoon sessions. If workshop timing meaningfully influences participant responses or engagement, we would expect to observe systematic differences in treatment effects between these two groups.

We estimate our primary specification using the intent-to-treat (ITT) estimator with binary outcome variables to assess whether the workshop's impact varies by time of day. Results are presented in Table 35. The effects of treatment are consistent in direction and of comparable magnitudes, regardless of whether the workshops took place in the morning or afternoon. Lacking statistical significance for two policy outcomes in the morning sessions likely reflects limited statistical power due to a smaller sample size in that subgroup, constraining our ability to detect effects.

Table 35. Causal effects of workshop participation on support for costly climate policies by timing of the day

	**Immediate effect**		
	Flight ban	Beef tax	Meat-free canteen
**Panel A: Morning workshops**			
Treatment	0.03	0.13^{*}	$0.15^{\cdot}$
	(0.07)	(0.05)	(0.07)
Num. obs.	290	291	293
N Clusters	33	33	33
Adj. R^2	−0.02	0.05	0.05
Block FE	Yes	Yes	Yes
Covariates	No	No	No
Estimator	ITT	ITT	ITT
**Panel B: Afternoon workshops**			
Treatment	0.07^{**}	0.06^{*}	0.06^{*}
	(0.02)	(0.02)	(0.02)
Num. obs.	1510	1519	1525
N Clusters	134	134	134
Adj. R^2	0.07	0.13	0.05
Block FE	Yes	Yes	Yes
Covariates	No	No	No
Estimator	ITT	ITT	ITT

Note: Panel A displays the results only for workshops implemented in the morning. Panel B displays the results only for workshops implemented in the afternoon. We use an Intent-to-Treat estimator, experimental block fixed effects, and cluster-robust standard errors at the workshop level. Our outcomes are dummy measures of policy support, where responses are coded 1 if respondents show support for the policy, 0 otherwise. $^{***}p < 0.001$; $^{**}p < 0.01$; $^{*}p < 0.05$; $^{\cdot}p < 0.1$.

F.5 Leave-one-out sensitivity analysis

To assess the robustness of our findings and verify that they are not driven by any single university, we conduct a leave-one-out sensitivity analysis. Specifically, we re-estimate our main specification iteratively, each time excluding one university from the sample. This procedure is implemented for each of the three policy support outcomes: the beef tax, flying restrictions, and meat-free canteen. We use an intent-to-treat estimator with university- and experimental block-fixed effects, with clustered standard errors at the workshop level.

The results of this analysis confirm the overall stability of the estimated treatment effects. Across the various specifications, the magnitude and direction of the effects remain consistent, and the exclusion of any individual university does not substantially alter the substantive interpretation of the findings. In particular, we find that the exclusion of a single university does not result in a reversal of the direction of the treatment effect or a loss of statistical significance across all outcomes. These results increase our confidence in the internal validity of our findings and suggest that they are not idiosyncratically driven by localised implementation features or sample peculiarities at specific universities.

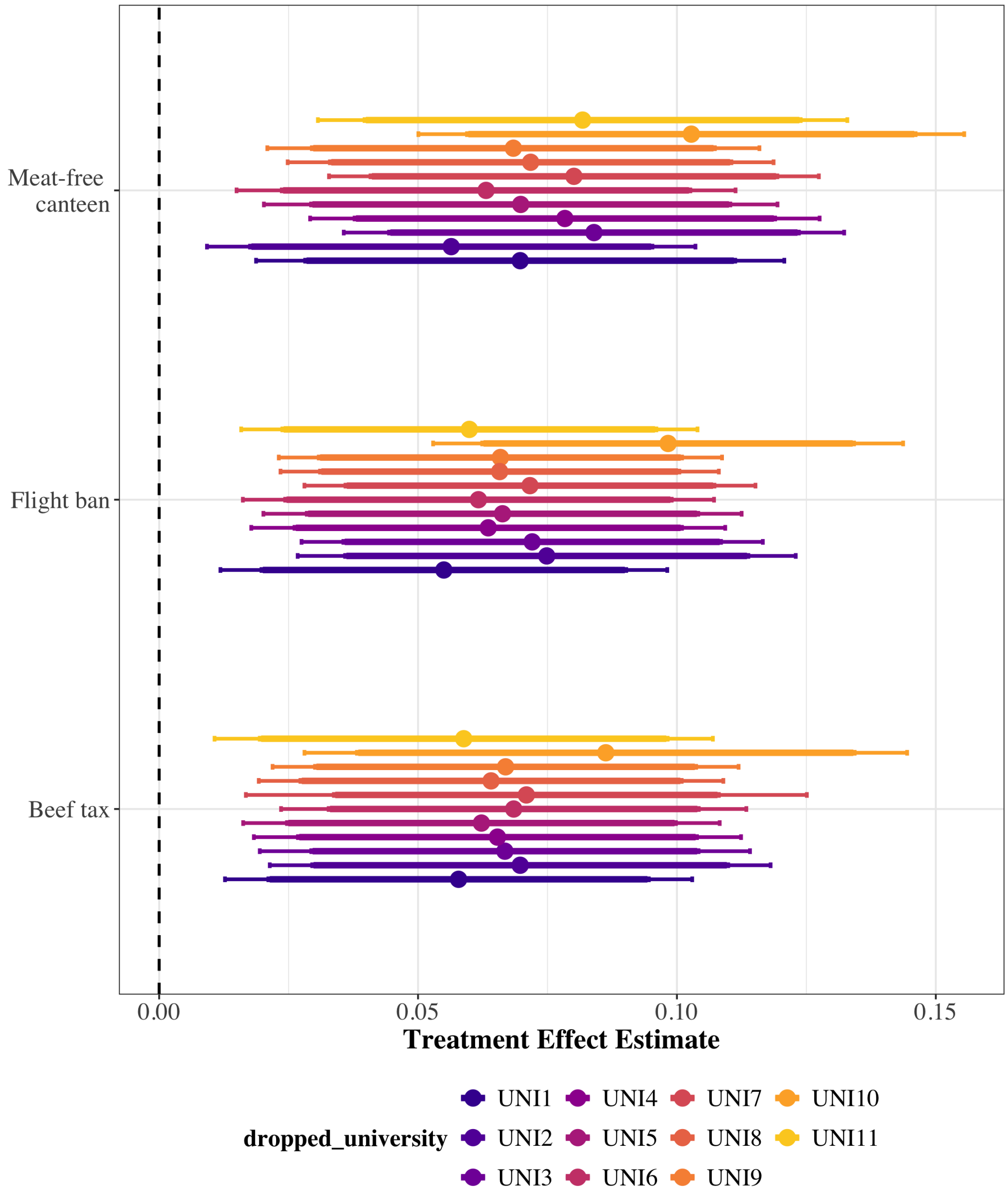

Figure 6. Leave-one-out sensitivity analysis. Estimates use a binary measure of policy support, where responses are coded 1 if respondents show support for the policy, 0 otherwise. Dots represents the ITT estimate using block randomisation fixed effects and cluster standard errors at the workshop level. Thick bars represent 90% confidence interval and thin bars the 95% confidence interval.

G Comparison of Effect Sizes

In this section we compare the observed effect of the 2tonnes on support for costly climate policies with other studies with similar designs and outcomes variables. Table 36 benchmarks the magnitude of our workshop's effect against a range of other experimental interventions which aim to shift attitudes and preferences of respondents through experimental interventions leveraging some form of persuasion, whether it be education and information provision, serious games, media framing, or in-person door-to-door canvassing. By selecting such a wide range of interventions, we can assess the impact of climate educational workshops like 2tonnes in relation to other, commonly used, forms of persuasion.

Because each study reports effects on different scales and outcomes, we compare them using Hedges' g, a standardized effect-size measure widely used in psychology and meta-analysis. Hedges' g expresses the treatment effect in standard deviation units, adjusting for sample size, which allows results from heterogeneous designs to be assessed via a common metric. These standardized effects are obtained by dividing the observed difference in an outcome measure between treatment and control groups by the pooled standard deviation of the outcome. For example, in our study we observe a consistent 7 percentage point effect on support for costly climate policies. This combined with a pooled SD of roughly 0.44 yields:

$g \approx \frac{0.07}{0.44} \approx 0.16.$

which is the value reported in Table 36 (g = 0.16, 95% CI [0.09, 0.23]).

Placed in context, this magnitude is the largest observed across the interventions compared here. It is comparable to the effect of scientific information provision reported by Diamond et al. (2020) [15] (g = 0.19), and substantially larger than the normative and messaging-based interventions from Vlasceanu et al. (2024) [16], which range from g = 0.03 for scientific consensus framing to g = 0.13 for a letter-to-future-generations intervention. Informational and media-based treatments generally cluster at the lower end of the distribution, with televised issue ads [17] yielding g = 0.04, consensus messaging [18] yielding g = 0.03, and door-to-door perspective-taking canvassing [19] yielding g = 0.00. The digital serious game evaluated by Sajjadi et al. (2022) [20] falls between these benchmarks at g = 0.09, though notably for a subgroup with less prior science education. This comparison highlights that the 2tonnes workshop produces an effect that is relatively large for experimental interventions targeting support for costly climate policies.

**Reference**	**Type of intervention**	**Policy outcome (support) studied**	**Hedges' g [95% CI]**	**N**
Diamond et al. 2020 [15]	Scientific information provision	Whether mitigating climate change should be a priority	0.19 [0.11, 0.26]	3000
**Our study**	**2tonnes workshop (3 h climate education)**	**Support for ban on flight, meat-free canteen, and beef tax**	0.16 [0.09, 0.23]	**1845**
Vlasceanu et al. 2024 [16]	Letter to future generation	Agreement on 9 climate policies	0.13 [0.09, 0.17]	59,440
Vlasceanu et al. 2024 [16]	Future self continuity	Agreement on 9 climate policies	0.11 [0.07, 0.15]	59,440
Sajjadi et al. 2022 [20]	Digital serious game vs. static website (MTurk)	Policy support regulating human impacts on Food-Energy-Water nexus	0.09 [0.03, 0.15]	152
Vlasceanu et al. 2024 [16]	Psychological distance	Agreement on 9 climate policies	0.06 [0.02, 0.10]	59,440
Vlasceanu et al. 2024 [16]	Dynamic social norms	Agreement on 9 climate policies	0.05 [0.01, 0.09]	59,440
van der Linden et al. 2015 [18]	Consensus-message experiment	Support for action	0.03 [−0.19, 0.24]	1104
Vlasceanu et al. 2024 [16]	Scientific consensus	Agreement on 9 climate policies	0.03 [0.01, 0.07]	59,440
Kalla and Broockman 2022 [17]	Televised issue ads	Support for LGBTQ and immigration policies	0.02 [−0.00, 0.05]	31,304
Broockman and Kalla 2016 [19]	Perspective-taking canvassing (10 min)	Support for nondiscrimination law	0.00 [−0.09, 0.09]	1825

Table 36. Summary of interventions and their effects on policy support (Hedges' g).

H Additional Experimental Elements

H.1 Framing Experiment – Main Survey

As part of the behavioral component of the main survey, we embedded a small framing experiment to test whether the way the NGO is described influences respondents' willingness to donate in the lottery task. Respondents were randomly assigned to one of three descriptions of the NGO: (1) a control frame stating that the NGO fights climate change, (2) a frame emphasising that the NGO reduces beef consumption, or (3) a frame stating that the NGO reduces air travel. These frames were shown immediately before respondents made their donation decision. The table below reports the estimated effects of these descriptions on donation amounts.

Overall, the framing manipulation produces limited changes in donation behavior, though the reduce beef consumption frame yields a modest reduction in donations relative to the control condition.

Table 37. Effect of NGO description on lottery donation

	**Lottery Donation**
Reduce air travel frame	-1.37
	(1.999)
Reduce beef consumption frame	$-3.20^{\dagger}$
	(1.828)
Treatment	$-2.55^{\dagger}$
	(1.461)
R^2	0.076
Adj. R^2	0.038
Num. obs.	1545
RMSE	30.91
N Clusters	167
Block FE	Yes
Estimator	OLS

Note: Estimates from ITT models with fixed effects for universities and experimental blocks. Standard errors are clustered at the workshop level (167 clusters). "Reduce air travel" and "Reduce beef consumption" are indicator variables for the two experimental descriptions of the NGO; the omitted (control) category is "Fight climate change." $^{***}p < 0.001$; $^{**}p < 0.01$; $^{*}p < 0.05$; $^{\dagger}p < 0.1$.

H.2 Endline Experiment – Meat in Canteen

As specified in our pre-analysis plan, we embedded a survey experiment in our endline survey. The aim was to examine whether information about in-group meat consumption affects support for costly climate policies, i.e., a meat-free university canteen. Additionally, we tested whether these frames affected the perceived fairness of all three costly climate policy outcomes. The motivation was to test whether social comparison or fairness considerations -— two mechanisms emphasised in the 2tonnes workshop -— could be activated through a brief informational treatment delivered six-weeks after the workshop concluded.

Respondents were assigned to one of three conditions:

- **T0 (Control):** No information.
- **T1 (Age-cohort in-group):** Respondents estimated how much meat 18–24-year-olds consume compared with older groups and were shown the correct information (young adults consume more meat).
- **T2 (National in-group):** Respondents estimated how much meat France consumes relative to the global average and were shown the correct statistic (twice the world average).

The experiment was administered after questions on the beef tax and flight ban, but before the question on support for a meat-free canteen, which serves as the primary outcome. We also measured fairness perceptions for all three policies on a 0–10 scale post-treatment.

Results

Table 38 reports the effects of the treatment on support for a meat-free university canteen. Neither informational treatment produced detectable shifts in policy support; estimates are close to zero and far from conventional significance thresholds. Table 39 examines effects on perceived fairness of the beef tax, flight ban, and canteen ban (Models 1–3). Again, the estimates are imprecisely estimated and do not reveal systematic treatment effects, although the coefficient for T2 in the canteen fairness model is positive and statitically significant the 90% level.

Taken together, the experiment does not yield evidence that brief informational prompts about in-group meat consumption meaningfully shift policy preferences or fairness perceptions in this sample.

The null findings are consistent with the low statistical power of the endline sample and the fact that all respondents had already participated in the workshop prior to receiving the framing treatment. As such, the experiment should be interpreted as exploratory, and the absence of detectable effects does not rule out meaningful social-comparison or fairness mechanisms in a better-powered design.

Table 38. Effect of Experimental Treatment on Support for Meat-Free Canteens

	**Meat-Free Canteen**
Treatment: Age-Cohort	−0.02
	(0.07)
Treatment: National	0.01
	(0.07)
R^2	0.20
Adj. R^2	0.03
Num. obs.	327
RMSE	0.49
N Clusters	123
Block FE	Yes
Estimator	OLS

Note: Estimates from OLS models with fixed effects for universities and experimental blocks. Standard errors are clustered at the workshop level (123 clusters). "Treatment: Age-cohort" and "Treatment: National" indicate assignment to the Age-Cohort and National In-Group informational treatments, respectively; the omitted reference category is the pure control (T0). $^{***}p < 0.001$; $^{**}p < 0.01$; $^{*}p < 0.05$; $^{\dagger}p < 0.1$.

Table 39. Effect of Experimental Treatment on Fairness Perceptions

	**Canteen Fairness**	**Beef Fairness**	**Flight Fairness**
Treatment: Age-cohort	0.53	-0.07	-0.10
	(0.47)	(0.27)	(0.36)
Treatment: National	$0.79^{\dagger}$	-0.11	-0.21
	(0.42)	(0.30)	(0.39)
R^2	0.147	0.233	0.171
Adj. R^2	-0.047	0.058	-0.017
Num. obs.	310	310	310
RMSE	3.10	2.42	2.56
N Clusters	121	121	121
Block FE	Yes	Yes	Yes
Estimator	OLS	OLS	OLS

Note: OLS models with university and experimental block fixed effects. Standard errors are clustered at the workshop level (121 clusters). "Treatment: Age-cohort" corresponds to the Age-Cohort in-group treatment; "Treatment: National" corresponds to the National in-group treatment; the omitted category is the pure control group. $^{***}p < 0.001$; $^{**}p < 0.01$; $^{*}p < 0.05$; $^{\dagger}p < 0.1$.

I Full Questionnaire

I.1 Introduction

This section provides the full survey questionnaire taken by participants in the 2tonnes workshop. It is administered via Qualtrics to participants either before or after taking the workshop. We provide the English language text of all questions and descriptions of measurement. The survey is distributed to participants in French. All questions are available in French upon request.

I.2 Demographics

d_age

- What is your age? [terminate if under 17]
  - Text Box

d_email

- Please enter your university email address.
  - Text Box

d_facilitator

- What is the facilitator name? (First & Last) [changes depending on context]
  - Text Box

d_before

- Are you filling out this survey at the beginning or the end of the 2tonnes workshop?
  - Before
  - After

d_female

- What is your gender?
  - Female

- Male
- Other
- Prefer not to say

d_rural

- Where did you grow up: in a rural or urban area? (If both, choose where you spent the majority of your years from the age of 10 to 18).
  - Rural
  - Peri-urban
  - Urban
  - Prefer not to say

d_income

- What is the total monthly income of your highest earning parent, after taxes?
  - <1.700€
  - <2.400€
  - <3.400€
  - <4.900€
  - >4.900€

d_party

- Which political party do you feel closest to?
  - Debout La France
  - Horizon
  - La France Insoumise (LFI)
  - Renaissance
  - Les Ecologistes – (EELV)
  - Les Républicains (LR)
  - Lutte Ouvrière (LO)
  - MoDem

  - Nouveau Parti Anticapitaliste (NPA)
  - Parti Communiste Français (PCF)
  - Parti Socialiste (PS)
  - Rassemblement Nationale (RN)
  - Reconquête (Eric Zemmour)
  - Other
  - None
  - I don't know
  - Prefer not to say

d_salience

- What do you think are the two most important issues facing France at the moment?
  - Crime
  - Economic situation
  - Rising prices/ inflation/ cost of living
  - Taxation
  - Unemployment
  - Terrorism
  - Housing
  - Government debt
  - Immigration
  - Health
  - The education system
  - Pensions
  - The environment and climate change
  - Energy supply
  - Other
  - None
  - Don't know

d_corrupt

- Please tell whether you agree or disagree with the following statement? – There is corruption in the national public institutions in France.
    - Measured on 5-point Likert scale: 1 (Completely Disagree) – 5 (Completely Agree)

d_beef

- Personally, in a typical week, how often do you eat red meat (including prepared meals)?
    - I am a vegetarian or a vegan
    - 1-2
    - 3-4
    - 5-6
    - 7+

d_fly

- How many times have you flown in the last 12 months in France or Europe? (A round trip corresponds to 2 journeys)
    - Text Box

d_psy

- To what extent do you feel that climate change will affect your life personally in the next 10 years?
    - Measured on a 5-point Likert scale: 1 (Not at all) – 5 (Extremely)

d_emotions

- When you think about climate change, what is your reaction? (Please choose up to 2 options).
    - Motivated
    - Anxious
    - Optimistic
    - Concerned
    - Indifferent
    - Calm

- Hopeful
- Guilty
- Sad
- Angry
- None of these emotions
- I don't know

d_participate

- Have you ever taken any of the following workshops before?
  - 2tonnes
  - Fresque du Climat
  - None of the above

d_cf

- Have you already calculated your carbon footprint using the 2tonnes tool?
  - Yes
  - No
  - Prefer not to say

d_activism

- Have you ever…? (Check all that apply)
  - Demonstrated for the environment or the climate
  - Demonstrated for another cause
  - Donated money to an environmental association or organization
  - Shared content with others to raise awareness of the climate and the environment
  - None of the above

I.3 Policies

p_beef – A high tax on beef products

- Imagine that, to combat climate change, the government decides to limit the consumption of beef products. For this purpose, a high tax on beef products is implemented, doubling their price. Do you agree or disagree with the following statements?.

  1. I am in favour of a high tax on beef products
  2. A high tax on beef products would be an effective means to combat climate change

- Both statements measured on a 5-point Likert Scale: 1 (Strongly Disagree) – 5 (Strongly Agree)

p_flying – A ban on domestic and international flights for locations reachable by train or bus within 6 hours

- Imagine that, to combat climate change, the government decides to limit flying. A ban on domestic and international flights to destinations accessible by train or bus within 6 hours is put in place. Do you agree or disagree with the following statements?

  1. I am in favour of implementing a ban on all flights between locations which are 6 hours apart via bus or train.
  2. Such a ban on flights would be an effective means to combat climate change.

- Both statements measured on a 5-point Likert Scale: 1 (Strongly Disagree) – 5 (Strongly Agree)

p_canteen – A ban on meat in the university canteen

- Would you be in favour of introducing exclusively vegetarian menus in the university canteen?
  - Measured on a 5-point Likert Scale: 1 (Very Unfavourably) – 5 (Very Favourably)

I.4 Knowledge

k_car

- Which of the following contributes the most to the carbon footprint of the average French citizen?

  - Car Usage
  - Meat Consumption
  - Airplane Travel
  - Heating
  - Use of digital and online services
  - I don't know

- The correct answer is "Car usage". Thus, measurement will be a binary variable which takes the value of 1 ("Car usage") and 0 (all other answers).

k__beef

- What is the estimated contribution of beef consumption to the carbon footprint of the average French person?
  - 3%
  - 15%
  - 30%
  - I don't know

- The correct answer is "15%". Thus, the measurement will be a binary variable which takes the value of 1 (15%) or 0 (all other answers)

I.5 Behavior

b__enough

- On a scale of 0 (not nearly enough) - 10 (more than enough), are the following actors currently doing enough to address climate change:
  - Individuals
  - Government
  - Firms/Private Business

- Each item is measured on a separate 0 to 10 scale which allows us to create two types of variable – a) simple numerical variable re-scaled from 0 to 1; b) perception gap variables where we subtract one actor's score from that of another.

p__lottery

- By completing this questionnaire and a follow-up questionnaire which you will receive in one month, you can enter a lottery to win 100 euros. Would you like to participate? (Results by March 1, 2025)
  - Yes
  - No

p_donation (Only shown to participants who select YES at p_lottery)

- If you win the lottery, would you be willing to donate part or all of your 100 euros to the climate NGO Réseau Action Climat, an organization which is [*insert additional information here depending on treatment described below*]? Please indicate the amount of your donation on a slider from 0 (nothing) to 100 (all).
  - Measured on a slider scale from 0 to 100

p_donation – different text to insert in question

- committed to fighting climate change
- committed to reducing beef consumption
- committed to reducing air travel

I.6 Workshop Feedback

w_enjoyed

- On a scale of 0 (did not enjoy) - 10 (enjoyed a lot), did you enjoy the workshop?

w_interview

- Would you be willing to be contacted in order to participate in a short interview about your experience taking the 2tonnes workshop?

References

[1] Vanessa Marie-Jane Aeschbach, Martin Schwichow, and Werner Rieß. Effectiveness of climate change education—a meta-analysis. *Frontiers in Education*, 10, May 2025. ISSN 2504-284X. doi: 10.3389/feduc.2025.1563816.

[2] Juliette N. Rooney-Varga, Rachel L. Coleman, Andrew P. Jones, Florian Kapmeier, Peyton Newsome, Krystal Noiseux, Bethany Patten, Kenneth Rath, and John D. Sterman. Interactive role-play with climate policy simulation can motivate evidence-based climate action. *Communications Earth & Environment*, 6(1):769, September 2025. ISSN 2662-4435. doi: 10.1038/s43247-025-02744-w.

[3] Helen Landmann, Wiebke Ziegler, and Robert Gaschler. Simulating climate policies influences how laypersons evaluate the effectiveness of climate protection measures. *Environment, Development and Sustainability*, May 2024. ISSN 1573-2975. doi: 10.1007/s10668-024-05028-z.

[4] Julia Cagé and Thomas Piketty. *A History of Political Conflict: Elections and Social Inequalities in France, 1789–2022*. Harvard University Press, Cambridge, Massachusetts, 2025. ISBN 978-0-674-24843-4.

[5] Adeline Lo, Jonathan Renshon, and Lotem Bassan-Nygate. A Practical Guide to Dealing with Attrition in Political Science Experiments. *Journal of Experimental Political Science*, 11(2): 147–161, 2024. ISSN 2052-2630, 2052-2649. doi: 10.1017/XPS.2023.22.

[6] Alexander Coppock, Alan S. Gerber, Donald P. Green, and Holger L. Kern. Combining Double Sampling and Bounds to Address Nonignorable Missing Outcomes in Randomized Experiments. *Political Analysis*, 25(2):188–206, April 2017. ISSN 1047-1987, 1476-4989. doi: 10.1017/pan.2016.6.

[7] Jonathan Rogers. Nothing to Lose: Charitable Donations as Incentives in Risk Preference Measurement. *Journal of Experimental Political Science*, 4(1):34–56, April 2017. ISSN 2052-2630, 2052-2649. doi: 10.1017/XPS.2017.4.

[8] Catherine C. Eckel and Philip J. Grossman. Altruism in Anonymous Dictator Games. *Games and Economic Behavior*, 16(2):181–191, October 1996. ISSN 0899-8256. doi: 10.1006/game.1996.0081.

[9] Eric Bettinger and Robert Slonim. Using experimental economics to measure the effects of a natural educational experiment on altruism. *Journal of Public Economics*, 90(8):1625–1648, September 2006. ISSN 0047-2727. doi: 10.1016/j.jpubeco.2005.10.006.

[10] James Habyarimana, Macartan Humphreys, Daniel N. Posner, and Jeremy M. Weinstein. Why Does Ethnic Diversity Undermine Public Goods Provision? *The American Political Science Review*, 101(4):709–725, 2007. ISSN 0003-0554.

[11] Lars Ivar Oppedal Berge, Kjetil Bjorvatn, Simon Galle, Edward Miguel, Daniel N. Posner, Bertil Tungodden, and Kelly Zhang. How Strong are Ethnic Preferences?, November 2015.

[12] Samantha Gonsalves Wetherell and Anna Josephson. The Cost of Climate Action: Experimental Evidence on the Impact of Climate Information on Charitable Donations to Climate Activism, September 2024.

[13] Kosuke Imai, Luke Keele, and Dustin Tingley. A general approach to causal mediation analysis. *Psychological Methods*, 15(4):309–334, 2010. ISSN 1939-1463.

[14] Mary Ryan Baumann, Monica Taljaard, Patrick J. Heagerty, Michael O. Harhay, Guangyu Tong, Rui Wang, and Fan Li. A tutorial on conducting sample size and power calculations for detecting treatment effect heterogeneity in cluster randomized trials with linear mixed models, November 2025.

[15] Emily Diamond, Thomas Bernauer, and Frederick Mayer. Does providing scientific information affect climate change and GMO policy preferences of the mass public? Insights from survey experiments in Germany and the United States. *Environmental Politics*, 29(7):1199–1218, November 2020. ISSN 0964-4016. doi: 10.1080/09644016.2020.1740547.

[16] Madalina Vlasceanu, Kimberly C Doell, Joseph B Bak-Coleman, Boryana Todorova, Michael M Berkebile-Weinberg, Samantha J Grayson, Yash Patel, Danielle Goldwert, Yifei Pei, Alek Chakroff, Ekaterina Pronizius, Sara Constantino, Michael J Morais, Philipp Schumann, Steve Rathje, Ke Fang, Salvatore Maria Aglioti, Mark Alfano, Andy J Alvarado-Yepez, Angélica Andersen, Frederik Anseel, Matthew A J Apps, Chillar Asadli, Fonda Jane Awuor, Flavio Azevedo, Piero Basaglia, Jocelyn J Bélanger, Sebastian Berger, Paul Bertin, Michał Białek, Olga Bialobrzeska, Michelle Blaya-Burgo, Daniëlle N M Bleize, Simen Bø, Lea Boecker, Paulo S Boggio, Sylvie Borau, Björn Bos, Ayoub Bouguettaya, Markus Brauer, Cameron Brick, Tymofii Brik, Roman Briker, Tobias Brosch, Ondrej Buchel, Daniel Buonauro, Radhika Butalia, Héctor Carvacho, Sarah A E Chamberlain, Hang-Yee Chan, Dawn Chow, Dongil Chung, Luca Cian, Noa Cohen-Eick, Luis Sebastian Contreras-Huerta, Davide Contu, Vladimir Cristea, Jo Cutler, Silvana D'Ottone, Jonas De Keersmaecker, Sarah Delcourt, Sylvain Delouvée, Kathi Diel, Benjamin D Douglas, Moritz A Drupp, Shreya Dubey, Jānis Ekmanis, Christian T Elbaek, Mahmoud Elsherif, Iris M Engelhard, Yannik A Escher, Tom W Etienne, Laura Farage, Ana Rita Farias, Stefan Feuerriegel, Andrej Findor, Lucia Freira, Malte Friese, Neil Philip Gains, Albina Gallyamova, Sandra J Geiger, Oliver Genschow, Biljana Gjoneska, Theofilos Gkinopoulos, Beth Goldberg, Amit Goldenberg, Sarah Gradidge, Simone Grassini, Kurt Gray, Sonja Grelle, Siobhán M Griffin, Lusine Grigoryan, Ani Grigoryan, Dmitry Grigoryev, June Gruber, Johnrev Guilaran, Britt Hadar, Ulf J J Hahnel, Eran Halperin, Annelie J Harvey, Christian A P Haugestad, Aleksandra M Herman, Hal E Hershfield, Toshiyuki Himichi, Donald W Hine, Wilhelm Hofmann, Lauren Howe, Enma T Huaman-Chulluncuy, Guanxiong Huang, Tatsunori Ishii, Ayahito Ito, Fanli Jia, John T Jost, Veljko Jovanović, Dominika Jurgiel, Ondřej Kácha,

Reeta Kankaanpää, Jaroslaw Kantorowicz, Elena Kantorowicz-Reznichenko, Keren Kaplan Mintz, Ilker Kaya, Ozgur Kaya, Narine Khachatryan, Anna Klas, Colin Klein, Christian A Klöckner, Lina Koppel, Alexandra I Kosachenko, Emily J Kothe, Ruth Krebs, Amy R Krosch, Andre P M Krouwel, Yara Kyrychenko, Maria Lagomarsino, Claus Lamm, Florian Lange, Julia Lee Cunningham, Jeffrey Lees, Tak Yan Leung, Neil Levy, Patricia L Lockwood, Chiara Longoni, Alberto López Ortega, David D Loschelder, Jackson G Lu, Yu Luo, Joseph Luomba, Annika E Lutz, Johann M Majer, Ezra Markowitz, Abigail A Marsh, Karen Louise Mascarenhas, Bwambale Mbilingi, Winfred Mbungu, Cillian McHugh, Marijn H C Meijers, Hugo Mercier, Fenant Laurent Mhagama, Katerina Michalakis, Nace Mikus, Sarah Milliron, Panagiotis Mitkidis, Fredy S Monge-Rodríguez, Youri L Mora, David Moreau, Kosuke Motoki, Manuel Moyano, Mathilde Mus, Joaquin Navajas, Tam Luong Nguyen, Dung Minh Nguyen, Trieu Nguyen, Laura Niemi, Sari R R Nijssen, Gustav Nilsonne, Jonas P Nitschke, Laila Nockur, Ritah Okura, Sezin Öner, Asil Ali Özdoğru, Helena Palumbo, Costas Panagopoulos, Maria Serena Panasiti, Philip Pärnamets, Mariola Paruzel-Czachura, Yuri G Pavlov, César Payán-Gómez, and Adam R Pearson. Addressing climate change with behavioral science: A global intervention tournament in 63 countries. *Science AdVAnceS*, 2024.

[17] Joshua L. Kalla and David E. Broockman. "Outside Lobbying" over the Airwaves: A Randomized Field Experiment on Televised Issue Ads. *American Political Science Review*, 116(3): 1126–1132, August 2022. ISSN 0003-0554, 1537-5943. doi: 10.1017/S0003055421001349.

[18] Sander L. van der Linden, Anthony A. Leiserowitz, Geoffrey D. Feinberg, and Edward W. Maibach. The Scientific Consensus on Climate Change as a Gateway Belief: Experimental Evidence. *PLOS ONE*, 10(2):e0118489, February 2015. ISSN 1932-6203. doi: 10.1371/journal.pone.0118489.

[19] David Broockman and Joshua Kalla. Durably reducing transphobia: A field experiment on door-to-door canvassing. *Science*, 352(6282):220–224, April 2016. doi: 10.1126/science.aad9713.

[20] Pejman Sajjadi, Mahda M. Bagher, Jessica G. Myrick, Joseph G. Guerriero, Timothy S. White, Alexander Klippel, and Janet K. Swim. Promoting systems thinking and pro-environmental policy support through serious games. *Frontiers in Environmental Science*, 10, October 2022. ISSN 2296-665X. doi: 10.3389/fenvs.2022.957204.
